# Systematic interrogation of mutation groupings reveals divergent downstream expression programs within key cancer genes

**DOI:** 10.1186/s12859-021-04147-y

**Published:** 2021-05-06

**Authors:** Michal R. Grzadkowski, Hannah D. Holly, Julia Somers, Emek Demir

**Affiliations:** Department of Molecular and Medical Genetics, Oregon Health & Science University, Portland, OR USA

**Keywords:** Cancer, Transcriptomics, Machine learning, Genomic variants, Drug response

## Abstract

**Background:**

Genes implicated in tumorigenesis often exhibit diverse sets of genomic variants in the tumor cohorts within which they are frequently mutated. For many genes, neither the transcriptomic effects of these variants nor their relationship to one another in cancer processes have been well-characterized. We sought to identify the downstream expression effects of these mutations and to determine whether this heterogeneity at the genomic level is reflected in a corresponding heterogeneity at the transcriptomic level.

**Results:**

By applying a novel hierarchical framework for organizing the mutations present in a cohort along with machine learning pipelines trained on samples’ expression profiles we systematically interrogated the signatures associated with combinations of mutations recurrent in cancer. This allowed us to catalogue the mutations with discernible downstream expression effects across a number of tumor cohorts as well as to uncover and characterize over a hundred cases where subsets of a gene’s mutations are clearly divergent in their function from the remaining mutations of the gene. These findings successfully replicated across a number of disease contexts and were found to have clear implications for the delineation of cancer processes and for clinical decisions.

**Conclusions:**

The results of cataloguing the downstream effects of mutation subgroupings across cancer cohorts underline the importance of incorporating the diversity present within oncogenes in models designed to capture the downstream effects of their mutations.

**Supplementary Information:**

The online version contains supplementary material available at 10.1186/s12859-021-04147-y.

## Background

Each tumor faces a common set of obstacles arising from internal dynamics and external defense mechanisms [1]. Tumor cohorts, however, are replete with diverse yet recurrent tactics for overcoming these shared obstacles. Tumorigenesis can thus be perceived as a landscape within which each tumor navigates a unique, multidimensional path, weaving between segments trodden by other tumors. A number of the early breakthroughs in cancer treatment directly resulted from coarse demarcations of these paths into distinct subtypes based on “landmarks”—usually defined by mutations and/or markers derived from proteomic or transcriptomic data—that were then used to engineer subtype-specific treatments [2–4].

Although these biomarker-based treatment matching criteria have proven effective in some precision medicine applications, there is a sizable subset of patients whose tumors harbor no discernible drug vulnerabilities, thus diminishing their likelihood of successful treatment and of patient survival [5–8]. Despite recent efforts to profile tumorigenic landmarks, for most such events we still know little about the downstream programs they trigger. As a result, most clinical guidelines depend on only a limited subset of specific variants within a gene or on other coarse biomarker-based demarcations [9, 10]. A clearer discernment of the downstream programs associated with mutations in cancer genes is thus a crucial prerequisite for addressing the challenges presently faced by precision oncology [11, 12].

Mutations of frequently altered genes often manifest as patterns of differential expression in downstream genes. Such patterns are usually referred to as the mutation’s transcriptomic signature or program. It was previously shown that it is possible to detect transcriptomic signatures for common cancer drivers by training machine learning algorithms to predict which samples in a tumor cohort harbor mutations [13–16]. A corollary to these results is that these mutation classifiers should also provide insight into the effects of the mutation in question. This hypothesis is supported by the correlations observed between these models’ predictions and other indicators of downstream activity including protein levels, response to drug treatment, and mutations in genes belonging to related cancer pathways [17, 18].

Further development of transcriptomic signatures is complicated by the dissimilitude of driver mutations within a gene [19, 20]. Although genes such as BRAF carry one hotspot responsible for almost all mutations observed in the gene in tumor cohorts [21], many genes implicated in tumor progression and proliferation have a widely distributed pattern of genomic alterations [22–25]. These mutations have varying degrees of impact; moreover, it is not uncommon for different alterations within a gene to carry out diametrically opposite roles in cancer development depending on context [26]. In cases such as KRAS this property has already been exploited to engineer clinical interventions targeted to a specific KRAS hotspot rather than the gene as a whole [27].

Can we measure these variable and divergent impacts? Consider the case of a gene with multiple mutation subsets, each significantly divergent from the rest with respect to downstream impact. With sufficient statistical power, we would expect transcriptomic classifiers trained to predict the presence of mutations within these subsets to be more accurate than a gene-wide classifier tasked with recognizing the presence of any mutation of the gene. Conversely, if we do not observe increased predictive performance for mutation subgroupings, it is likely that they are convergent, or at least share convergent characteristics identifiable by a classification model. Although subgrouping-specific classifiers benefit to a certain degree from having a more uniform set of downstream effects to identify in a tumor cohort, they must also overcome the loss in statistical power inherent in characterizing a subset of the available mutated samples. The discovery of mutation groupings robustly associated with better-performing classifiers within a gene would hence clearly present strong evidence of divergence.

For a given gene, the landscape of transcriptomic classifier accuracy across mutation subgroupings should thus inform us about the convergent and divergent effects of these genes’ mutations. This is useful for two immediate clinical purposes: estimating the likelihood that a variant of unknown significance has an effect similar to a previously characterized hotspot variant, and obtaining informed stratification criteria for recurrent mutations to aid in the design of clinical trials and precision medicine guidelines. Based on these observations, we interrogated frequently altered genes in large tumor cohorts for mutation subgroupings associated with improved classification performance. Instead of focusing on a single gene or pathway of interest, we sought to create a framework inspired by class-grouping approaches [28–30] which would generalize well to the population of somatic alterations recurrent in cancer. Specifically, we tested 12,871 groupings of mutations selected by applying a hierarchical class-grouping search heuristic to the mutation profiles of 200 cancer genes across 17 tumor cohorts. This allowed us to confirm previous findings showing that it is possible to predict the presence of mutations associated with cancer using regression models trained on expression data and to demonstrate that the mutation profiles of a multitude of cancer genes can be better characterized using our subgrouping-specific models.

## Results

### Enumerating cancer gene subgroupings in a tumor cohort

We first applied our subgrouping framework to the METABRIC cohort, which contains 1499 breast cancer tumor samples with both expression and mutation calls [31]. Applying UMAP, a manifold-based unsupervised learning technique [32], to the expression profiles of these samples revealed clusters corresponding to molecular subtypes known to have unique transcriptomic profiles [33, 34] (Additional file 1: Figure S1). To ensure that this heterogeneity at the molecular level did not confound our interrogation of heterogeneity at the genomic level we filtered out samples not belonging to luminal A, the most prevalent subtype. This resulted in a more uniform cohort consisting of 1017 tumor samples hereafter referred to as METABRIC-(LumA).

We identified oncogenic and tumor suppressor genes based on their inclusion in the OncoKB database [35]. Our search for mutation groupings was restricted to the subset of these genes with point mutations present in at least 20 samples in the cohort where the term *point mutation* is applied loosely to describe any genomic alteration involving a small number of nucleotides (e.g. SNPs, frameshifts, inframe insertions, etc.) while the more general term *mutation* is reserved for the broader collection of genomic variants spanning both point mutations and large-scale mutations such as copy number alterations (CNAs).

For each gene, we enumerated subsets of its point mutations present in METABRIC-(LumA) that could potentially have a biologically meaningful downstream transcriptomic signature. This was done by first hierarchically organizing mutations according to attributes particularly likely to have a bearing on their downstream effects. For example, the 488 samples carrying any point mutation of PIK3CA in METABRIC-(LumA) can be decomposed according to the exon the mutation falls on: 248 samples for the 21st exon, 177 for the 10th exon, 45 for the 5th exon, and so on. These partitions can be further subdivided according to the codon location of each mutation: 226 samples for exon 21 and codon 1047, 109 for exon 10 and codon 545, 56 for exon 10 and codon 542, 40 for exon 5 and codon 345, etc. We can thus create a gene-wide classification task in which a prediction algorithm must separate the 488 PIK3CA mutants from the 529 PIK3CA wild-types in the METABRIC-(LumA) cohort, as well as subgrouping classification tasks in which we seek to separate e.g. the 109 samples with PIK3CA mutations on exon 10, codon 545 from the 908 samples wild-type for {PIK3CA:exn10:cdn545} in the METABRIC-(LumA) cohort.

The above example organizes PIK3CA mutants into a tree according to genomic location: we first create branches corresponding to exons, and then add a further layer of branches corresponding to codons. Constructing subgrouping classification tasks using these branches thus leverages the expectation that PIK3CA mutations close to one another are more likely to have similar downstream effects, while allowing us to search over different scales (exon vs. codon) at which this prior might be true. This framework can be extended to any other combination of available mutation attributes; for the purposes of this study we focused on four combinations particularly likely to produce biologically meaningful subgroupings. The first of these is the exon to codon hierarchy introduced above with a further layer specifying the particular codon that replaces the wild-type: $\big \langle$Exon $\rightarrow$Codon Position $\rightarrow$Codon Substitution$\big \rangle$. On the other hand, $\big \langle$Consequence $\rightarrow$Exon$\big \rangle$ first groups together mutations with the same effect on the protein sequence (missense, stop lost, frameshift, etc.) and then further subdivides according to exonic location. This accounts for the possibility that, within particular genes, mutations of the same type are more likely to have similar downstream effects than mutations that are close to one another. Finally, both $\big \langle$SMART Domain $\rightarrow$Consequence$\big \rangle$ and $\big \langle$Pfam Domain $\rightarrow$Consequence$\big \rangle$ first organize mutations according to their overlap with a known protein domain and then segregate mutations according to downstream effect. These two trees enhance how the “closeness” of mutations is defined using a secondary source of information about structural units within the protein they affect while also incorporating information on the general nature of the type of genomic aberration caused by the mutation.

We generated subgroupings using this collection of mutation trees by searching over possible tree branches whose mutations were present in at least twenty samples in METABRIC-(LumA), thus providing enough positive examples for a classifier to be trained and tested on. A subgrouping branch could thus be one of the leaves of a tree, i.e. {PIK3CA:exn21:cdn1047:H1047R}, or one of the internal branches spanning multiple leaves, i.e. {PIK3CA:exn21}. We further expanded our search to include subgroupings corresponding to a combination of two such tree branches, each containing at least ten mutated samples in the cohort, with the resulting subgrouping including all samples carrying a mutation on either of the branches. In METABRIC-(LumA) this produced a total of 772 subgroupings across the 38 cancer genes with at least 20 mutated samples, of which 217 were subgroupings of PIK3CA, 105 were subgroupings of TP53, and 101 were subgroupings of GATA3 (see Additional file 2: Tables S1 for a complete list of enumerated subgroupings).

### Training and evaluating subgrouping transcriptomic signatures

For each gene we trained a classifier to predict which samples in the cohort carried at least one point mutation on the gene—we refer to this as the gene-wide task. We then trained separate classifiers to predict which samples carried individual subgroupings’ mutations, referred to as the set of subgrouping tasks. Each task involved applying a logistic regression classifier utilizing the ridge regularization penalty to the given cohort’s expression data in order to produce sample scores that corresponded to the model’s confidence that they harbored a mutation in the subgrouping (see “Methods” section) [36]. The logistic ridge regression model accomplished this by identifying a set of coefficients, one for each gene feature in the expression data, such that features with large positive coefficients were robustly associated with up-regulation by the task’s mutations while features with large negative coefficients were associated with down-regulation.

Model tuning and training was done using ten iterations of four-fold cross-validation; a classification task thus generated ten scores for each cohort sample. We measured a classifier’s ability to identify a transcriptomic signature for its assigned task using the area under the receiver operating characteristic curve metric (AUC) calculated using samples’ mean scores across the ten cross-validation iterations. A task AUC of 1.0 thus signified “perfect” classifier performance in which all samples carrying mutations in the subgrouping had higher mean scores than the mean score of any sample that was wild-type with respect to the subgrouping, while an AUC of 0.5 signified “random” performance in which the mean score of a subgrouping-mutant sample was equally likely to be lower or higher than the mean score of a wild-type sample.

To compare the performances of two different classification tasks, we utilized the AUCs specific to individual cross-validation iterations. As the same forty training-testing tumor sample splits were used for a cohort’s ten cross-validation iterations across all of its experiments, classifier performances on each iteration could be compared directly. We calculated ten “cv” AUCs for a task, each using samples’ predicted scores from one cv iteration as opposed to the average across all ten for the original task AUC. In our analyses we report the difference between two task AUCs as “cv-significant” only if all ten comparisons between their respective cv AUCs yielded a disparity in the same direction. In particular, we highlight genes in which at least one mutation subgrouping classifier’s performance met this criterion positively relative to the classifier trained on the gene-wide task—that is, if the subgrouping classifier had a higher “cv” AUC than the gene-wide classifier in all ten direct comparisons.

### Characterizing mechanisms underlying divergent subgrouping performance

Our approach relies on manipulating the labels to be predicted in a classification task by changing some of the “mutated” sample labels to “wild-type”, with the expectation that there are at least some genes in which the original labeling does not accurately reflect the diversity of downstream effects wrought by their mutations. However, an increase in AUC as a result of such a manipulation can happen for several different reasons. If some of the gene’s mutations are inactive, then treating samples carrying these mutations as wild-type should result in a subgrouping classifier similar to the gene-wide classifier, albeit applied to a task with more accurate labels. On the other hand, if some of the gene’s mutations are divergent by the type of downstream effect they cause, and not just by whether they cause a downstream effect in the first place, then we would expect the behaviour of the corresponding subgrouping classifier to be much more distinct from its gene-wide counterpart.

To quantify these types of differences between subgrouping classifiers and gene-wide classifiers, and thus provide a more complete explanation for observed improvements in AUCs, we created a metric capturing how much more accurately a given subgrouping classifier predicted the presence of mutations in its subgrouping relative to the gene-wide model’s performance on the same set. If a subgrouping simply excludes inactive mutations of the gene, then we would expect the gene-wide classifier to also tend to correctly classify subgrouping-mutated samples as “mutant”, and be penalized for being forced to treat the other mutations of the gene as also “mutant”. It follows that if we instead observe that the gene-wide classifier is more likely to err in predicting subgrouping mutations, then the subgrouping classifier is divergent not just in performance but also in finding a novel way of separating subgrouping-mutated samples from the remaining samples in the cohort. We therefore compared the AUC calculated using the gene-wide classifier’s predicted sample labels on the subgrouping task to the subgrouping classifier’s task AUC originally calculated on the same labels. Unlike comparisons of AUCs discussed above, this comparison involves two models’ predictions on the same set of labels, and thus we can apply DeLong’s test to obtain a *p* value for whether the subgrouping classifier does a better job of discerning the presence of subgrouping-mutated samples than the gene-wide classifier [37]. We report this *p* value where applicable in the text below using the label $p_{Divg}$ to quantify the degree to which using a subgrouping instead of a gene-wide classifier results in a novel classification of a cohort’s samples rather than removing false positives from the gene-wide task.

### Subgrouping classifiers uncover alteration divergence in a breast cancer cohort

**Fig. 1:**
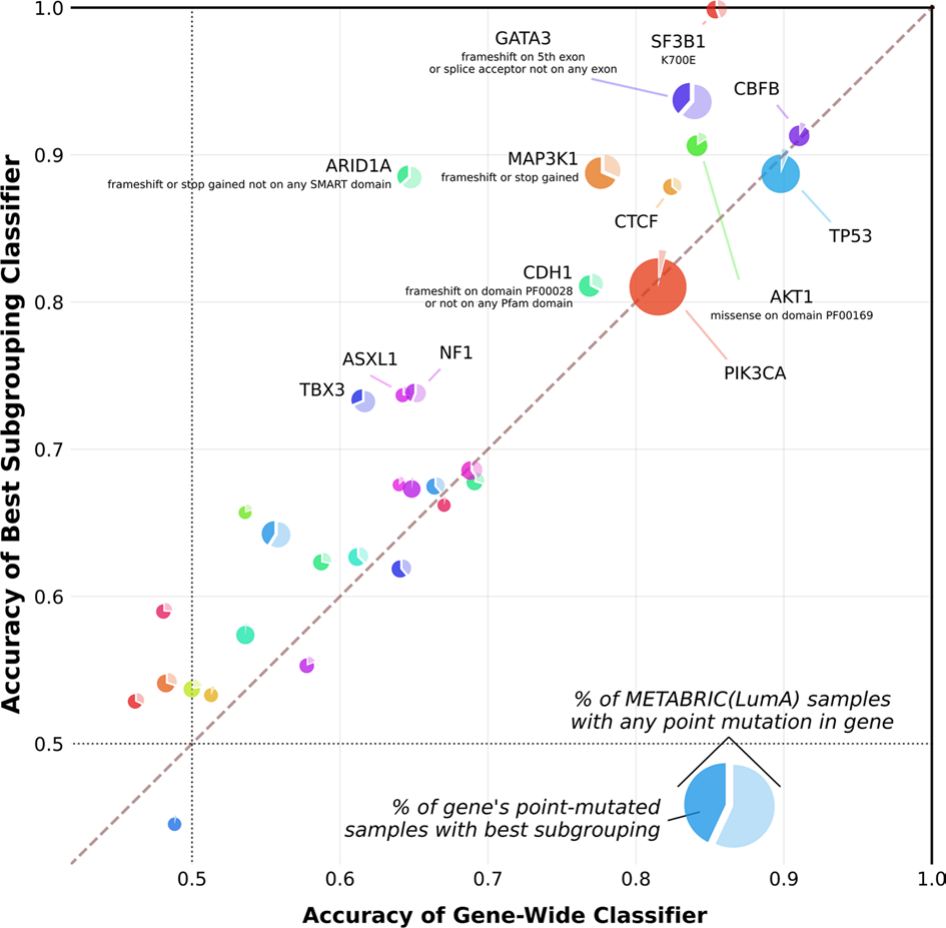
Divergent transcriptomic programs are a recurring feature of frequently mutated genes in breast cancer. 772 subgroupings within the point mutations of 38 genes having known links to cancer processes in METABRIC-(LumA) were enumerated by grouping together variants with shared properties. A logistic ridge regression classifier was trained to predict the presence of any point mutation in each of these genes as well as the presence of each enumerated subgrouping. Comparing the classification performance (AUC) for each gene-wide task (x-axis) to the best performance across all tested subgroupings of the gene (y-axis) reveals subgroupings within genes such as GATA3 and MAP3K1 with downstream effects that are consistently separable from the remaining mutations of the gene. The pie charts’ areas are proportional to the number of samples in the cohort that carry any point mutation of the corresponding gene; the darker slice inside each pie is scaled according to the proportion of these samples carrying a mutation in the best subgrouping. A gene label is included wherever the AUC of the best task exceeded 0.7; a description of the best subgrouping is also included wherever its task performance was cv-significantly higher than that of its gene-wide counterpart. Six genes in which no subgroupings were found have been omitted from this plot. The corresponding plots for the other cohorts used for training in this study can be found at Additional file 12: Figure S11

The transcriptomic signatures trained on the 810 gene-wide and subgrouping tasks enumerated in METABRIC-(LumA) revealed that many of the frequently mutated cancer genes in breast cancer have readily identifiable downstream expression effects. Crucially, a sizeable subset of these genes contain subgroupings with expression signatures that diverge from those associated with the gene as a whole (Fig. 1). For example, while it is already easy to find a downstream effect in the expression data for GATA3 point mutations when they are considered as a whole (177 mutated samples; AUC = 0.840), there are several GATA3 subgroupings that produced even more accurate transcriptomic signatures. Among the 26 found to be cv-significant with respect to the gene-wide task were all splice variants (47 samples; AUC = 0.916; $p_{Divg}$: 0.018), frameshifts overlapping the GATA3 Pfam zinc finger domain and all splice variants (72 samples; AUC = 0.920; $p_{Divg}$: 0.00028), as well as splice variants not assigned to an exon of GATA3 (44 samples; AUC = 0.925; $p_{Divg}$: $6.0\times 10^{-5}$). The subgrouping of GATA3 with the best classifier performance in METABRIC-(LumA) consisted of the frameshifts on the fifth exon combined with the splice variants not assigned to an exon (68 samples; AUC = 0.936; $p_{Divg}$: $1.4\times 10^{-4}$).

Clearly, GATA3 splice variants are not only divergent from most remaining GATA3 mutations, but share a similarity with some GATA3 frameshifts that is best characterized using our $\big \langle$ Consequence $\rightarrow$ Exon$\big \rangle$ mutation hierarchy. Furthermore, while samples carrying any type of GATA3 mutation evidently have expression profiles distinct from those of samples that are wild-type for GATA3, our experiment demonstrates that it is also possible to find signatures that are able to consistently differentiate types of mutations within the GATA3 mutation landscape. This is consistent with recent work showing that GATA3 mutations in breast cancer can be segregated by their effect on the function of the GATA3 protein into subsets consistent with those identified as divergent above [38]. Notably, splice site variants of GATA3 are observed almost exclusively in the luminal A but not in the luminal B subtype. This strongly suggests that either the splice variants drive cancer exclusively to luminal A or they are positively selected in luminal A. Both cases indicate the presence of a contextually-dependent functional divergence linked to the best-performing subgroupings found in our analysis.

Our approach was also able to identify cases in which truncating substitutions and frameshifts are clearly divergent from mutations much more likely to leave the structure of the protein intact. In MAP3K1, the best found subgrouping consisted solely of these two types of mutations and was also found to have an AUC cv-significantly higher than that of the MAP3K1 gene-wide task (102 samples out of 149 point mutants; AUC = 0.889; $p_{Divg}$: 0.012). The poor task performance of the subgrouping of nonsense mutations (28 samples; AUC = 0.688) suggests that the downstream effects of these mutations are much weaker than those of MAP3K1 frameshifts, though still more evident than the effects of MAP3K1 missenses (39 samples; AUC = 0.575). ARID1A was similar in that nonsense mutations and frameshifts were cv-significant compared to ARID1A mutations as a whole (26 samples; AUC = 0.878; $p_{Divg}$: 0.040), with the best subgrouping (also cv-significant) differing only in the omission of a single sample with a mutation overlapping a SMART domain (25 samples; AUC = 0.885; $p_{Divg}$: 0.010). This is consistent with the rarity of ARID1A missense mutations compared to nonsense mutations and frameshifts in other cancer types, suggesting that missense mutations are not selected for in general due to their lack of an effect on downstream processes [39, 40]. For CDH1, the only cv-significant subgrouping was composed of frameshifts overlapping the cadherin repeated Pfam domain and mutations not overlapping any Pfam domain (46 samples out of 68 point mutants; AUC = 0.812 vs. 0.770; $p_{Divg}$: 0.506), likely reflecting the subset of CDH1 mutations especially likely to cause the loss of E-cadherin implicated in particularly aggressive breast cancer tumors [41].

In line with expectations, genes such as AKT1 and SF3B1 whose mutation landscapes are dominated by a single hotspot featured divergent subgroupings incorporating the hotspot. Nevertheless, the remaining components of these subgroupings were instructive as to the uniqueness of these hotspots with regard to downstream effects relative to other mutations present on the same gene. In the former case, the E17K subgrouping exhibited better performance than the gene-wide task (48/64 AKT1 point mutants; AUC = 0.883 vs. AUC = 0.842; $p_{Divg}$: 0.814), but classifiers fared just as well treating all AKT1 missenses as a whole (57 samples; AUC = 0.885; $p_{Divg}$: 0.152). The best AKT1 subgrouping consisted of missenses falling within the Pleckstrin homology domain (54 samples; AUC = 0.908; $p_{Divg}$: 0.224); unlike the two aforementioned subgroupings its performance was also cv-significant compared to the gene-wide task. It is therefore likely that AKT1 mutations have similar regulatory consequences to E17K as long as they are in a position to disrupt binding processes. Given the lack of strong DeLong divergence relative to the gene-wide task for these subgroupings, we can also infer that the remaining AKT1 mutations lack a strong downstream effect. Likewise, in SF3B1 mutations on the 15th exon were found to be highly divergent (27/47 SF3B1 mutants; AUC = 0.999 vs. AUC = 0.855; cvSig; $p_{Divg}$: 0.022). In this case, improved classification performance was primarily due to the presence of the K700E hotspot which accounted for all but one of these mutants, and could also be predicted nearly perfectly on its own (26/47 SF3B1 mutants; AUC = 0.999; cvSig; $p_{Divg}$: 0.034). Moreover, the SF3B1 subgrouping which excluded silent mutations yielded a more modest boost in the quality of the transcriptomic signature (39/47 SF3B1 mutants, AUC = 0.928, cvSig; $p_{Divg}$: 0.232). This suggests that SF3B1 variants can be ordered by the strength of their downstream effects, with K700E mutations having the most significant impact, although the weak evidence of DeLong divergence leaves this conclusion ambiguous.

Among the 32 genes for which any subgroupings were enumerated in METABRIC-(LumA), these six (GATA3, MAP3K1, ARID1A, CDH1, AKT1, and SF3B1) were the only ones that had any subgroupings significantly divergent in performance from their gene-wide counterparts according to our cv-significance criterion. However, there were only four other genes with any classification tasks achieving an AUC of at least 0.8 (TP53, PIK3CA, CTCF, and CBFB). Our findings for TP53 were particularly surprising, as it has long been assumed that TP53 mutations can be divided into missense hotspots that result in gain-of-function and loss-of-function terminating substitutions and frameshifts [42]. However, recent literature suggests a more complicated relationship between these two classes of TP53 mutations, with many TP53 missense substitutions exhibiting an ability to disrupt the downstream activity of the TP53 protein in a dominant-negative fashion [43]. This suggests that our classification tasks are identifying a monolithic dysregylatory program in TP53 that combines the gain- and loss-of-function effects of its mutations in the absence of a cleanly delineated subset of TP53 mutations associated solely with one type of effect or the other.

Likewise, we did not find any subgroupings of PIK3CA in METABRIC-(LumA) with significantly better performance than the gene-wide classifier despite the wide variety of PIK3CA hotspots in the cohort, including N345K, E542K, and H1047R. This is concordant with work showing that while the mutated loci of PIK3CA exhibit varying downstream effects in proportion to their recurrence in tumor cohorts, these effects differ primarily in degree, not in kind [24]. In the case of CTCF, we were still able to find a subgrouping with considerably better performance than the gene-wide task (25/39 CTCF point mutants; AUC = 0.879 vs. 0.825). Although this task did not achieve cv-significance, it nevertheless suggests that CTCF contains divergent subgroupings discoverable using larger cohorts or wider subgrouping enumeration criteria. Finally, the lack of divergence among the 63 CBFB point mutants suggests that any of them are equally sufficient to disrupt the master-regulatory processes wrought by the gene [44].

Nevertheless, successfully training gene-wide classifiers for these well-known cancer drivers lends further credence to our framework’s ability to identify downstream effects where they would reasonably be expected to occur. It may be the case that nuanced yet consequential differences exist between the downstream expression effects associated with such genes, but that these differences are overshadowed by an expression program common to a sufficiently high proportion of the mutations. Similarly, whatever heterogeneity exists within the mutational profiles of these genes may be too granular to observe without access to larger tumor cohorts. Our results are thus better interpreted as proving the divergence inherent in particular downstream effect profiles rather than disproving the divergence of others. In the same vein, in three genes subgrouping tasks achieved an AUC of at least 0.7 (but less than 0.8) and exhibited evidence of divergence that was insufficient to pass our relatively strict cv-significance threshold (ASXL1: AUC = 0.738 vs. AUC = 0.643; NF1: AUC = 0.738 vs. AUC = 0.652; TBX3: AUC = 0.732 vs. AUC = 0.617).

The remaining nineteen genes in METABRIC-(LumA) with poorer classification performance most likely reflect the limitations of using transcriptomic cohorts to predict mutated states. It is possible that the presence of dominant driver mutations with strong downstream effects confounds our classifiers’ ability to quantify these effects for mutations with weaker downstream impact. Furthermore, although our data pre-processing workflow involved identifying and removing samples with clearly distinct transcriptomic profiles associated with molecular subtypes, our METABRIC-(LumA) cohort could be contaminated by subpopulations of samples that are more subtly aberrant, thus making it more difficult for a classifier to recognize the downstream signal of some mutations. These genes also tend to have a relatively small number of mutated samples (median point mutants: 31.5 vs. 68 for the other ten genes), which makes it more difficult for a classifier to robustly characterize the differences between the population of mutants and the population of wild-types in the METABRIC-(LumA) cohort, especially in the presence of the aforementioned confounding factors. Finally, in at least some of these cases the mutations in question are passengers and hence do not have significant downstream effects.

### Classifier coefficients identify regulatory mechanisms associated with divergent subgroupings

We sought to ground these comparisons of the performances of our subgrouping classifiers by also comparing the coefficients they assigned to gene expression features. Forty sets of coefficients were found for each classification task, one for each cross-validation fold used in training and testing; we henceforth report the average coefficient for each gene across these sets in a given task. We found this average easier to interpret than the coefficients of a individual logistic ridge regression model which searches for the most parsimonious set of weights such that predictive performance is maximized. Although a single model may not need to identify all of the expression features associated with the set of mutations in question in order to produce accurate output labels, it is far less likely that a gene whose expression is robustly associated with the set of mutations in question would be ignored by all forty trained classifiers.

The largest such coefficient in the gene-wide GATA3 task trained on METABRIC-(LumA) belonged to MEGF10 (Additional file 3: Figure S2), which has been previously implicated in cancer processes downstream of GATA3 [45]. MEGF10 was also weighted heavily in the best found subgrouping task, having the 37th largest absolute magnitude out of 13018 genes considered by the model. On the other hand, although neither KDM1B(LSD2) nor PRDM15 had sizeable coefficients in the best found subgrouping task, both were striking in their considerable contribution to the gene-wide task. This is intriguing as KDM1B plays an important role in breast cancer pluripotency and migration [46]. Although not much is known about the relationship between GATA3 and KDM1B, the paralogous KDM1A(LSD1)’s role in luminal breast cancer via GATA3 is well documented [47]. Furthermore, PRDM15 was recently identified as a gatekeeper for naive pluripotency [48]. Similarly to KDM1A, although not much is known about the relationship between PRDM15 and GATA3, family members PRDM2, ZFPM1 and ZFPM2 have been identified as GATA3 modulators [49, 50].

Hierarchical clustering of averaged coefficients across GATA3 mutation subgrouping tasks with an AUC of at least 0.7 revealed a number of expression programs that were differentially weighted across the subgroupings of GATA3 (Additional file 4: Figure S3). The best found subgrouping (splice acceptor not on any exon or frameshift on 5th exon) is seen in a clear cluster of subgroupings which exhibit negative coefficients for a block of genes that are weighted positively in the gene-wide task. Of the five genes in this block that had the greatest differences in coefficient magnitude between these two tasks, only NPAS4 has not been previously assessed in the context of human breast cancers (though it has recently been a focus of murine breast cancer PDX models [51]). The remaining four have all been studied for their effects in the development and maintenance of human breast cancers (TNP1 [52–54], FST [55], ANKRD40 [56], TRH [57, 58]).

These coefficients also helped to shed further light on the cases where we could not find any subgroupings with divergent classifier performance. TP53 subgroupings were almost universally associated with strong down-regulation of PHLDA3 and AEN, both of which are well-characterized targets of TP53 which play key roles in mediating the tumor suppressing functions of the p53 protein [59, 60]. Clustering of coefficient profiles across TP53 subgroupings revealed that subgroupings associated with divergent coefficients tended to have much lower classification performance, making it more likely that this divergence stems from model overfitting than actual impact on downstream cancer processes (Additional file 4: Figure S3). This lends further support to the hypothesis that the similarities between the transcriptomic effects of mutations of the p53 protein in luminal A breast cancer outweigh the differences.

PIK3CA subgroupings were even more homogeneous with respect to their logistic ridge regression model coefficients, with almost all subgroupings following a similar profile of up- and down-regulation as the gene-wide PIK3CA task, thus further supporting the lack of heterogeneity across the effects of PIK3CA mutations in breast cancer. Similar behavior observed in cases such as MAP3K1, SF3B1, AKT1, and CDH1 lends additional credence to the conclusion that the divergence with respect to subgrouping classifier performance in these genes is driven by the identification of active mutations by the best found subgroupings (Additional file 4: Figure S3).

### Comparison benchmarks contextualize subgrouping task performance

Our approach to characterizing transcriptomic heterogeneity within the alteration profiles of cancer genes is based on testing as many mutation subgroupings as possible to identify those with divergent expression signatures. Although we have already demonstrated that this strategy can be gainfully applied to find and characterize such subgroupings, it is also clearly susceptible to multiple hypothesis testing—how can we be sure that the improvements in AUC we have observed are not simply the upper tail of the noise inherent in measuring the accuracy of a large population of classifiers? Our prediction pipelines’ persistent ability to produce higher scores for samples in which a particular set of mutations is present leads us to claim that the set of mutations must have some biological relevance, but this relevance is difficult to establish without also comparing the classification performance against other sets of samples that could have been selected from the cohort to construct classification tasks.

We thus created a set of classification tasks to predict the presence of randomly chosen sets of samples of the same size as the mutation subgroupings we previously tested in METABRIC-(LumA), with five such tasks constructed for each subgrouping. The distribution of AUCs for this null background set of 4050 tasks was markedly lower than the corresponding distribution for tasks related to cancer genes, with a median AUC of 0.494 (Additional file 5: Figure S4). This confirmed that the mutation labels associated with cancer genes encode a significant amount of information relative to labels randomly assigned to the cohort. We also created an analogous gene-specific null background set of classification tasks by randomly selecting five size-matched subsets for each subgrouping from the collection of samples carrying any point mutation of the mutated gene. The performance observed for these tasks revealed that our hierarchical organization of genes’ mutations yielded better subgroupings than those that could be found by simply picking subsets of mutations occurring on the gene at random (Additional file 6: Figure S5). For example, only one of the 505 randomly chosen sets of GATA3 mutations managed an AUC even barely higher than that of the gene-wide task (AUC = 0.8402 vs. AUC = 0.8395), while 44.6% of our GATA3 subgroupings had higher AUCs than this “optimal” random GATA3 subgrouping. Five of the six genes with cv-significant subgroupings relative to the gene-wide task lacked any gene-specific random subgroupings which were cv-significant. In the case of the lone exception, MAP3K1, the best found subgrouping’s AUC was nevertheless cv-significantly higher than the AUC of any of the random subgroupings.

Since our method requires scanning a sizeable population of subgroupings, we opted to use a logistic ridge regression classifier that efficiently scales up to a large number of tasks. However, this choice of algorithm can potentially prevent us from detecting nonlinear transcriptomic signatures. Our tuning regime was also designed to be fairly straightforward in order to reduce computational load, testing only eight ridge regularization hyper-parameter values. To find whether our mutation prediction results in METABRIC-(LumA) were affected by these efforts to reduce the computational cost of our classification pipelines, we repeated the above experiment with radial basis function support vector [61] and random forest classifiers [62] as well as with a larger tuning grid for the logistic ridge regression classifier. We found that these more complex classifiers failed to produce improved AUC performance across our prediction tasks and did not affect the efficacy of subgrouping tasks relative to gene-wide tasks despite taking up to an order of magnitude longer to run to completion (Additional file 7: Figure S6A–C). The mutational profile heterogeneity we observe is thus not a by-product of the behavior specific to any one particular classification method, and can be observed using a relatively simple learning framework.

### Enlarging the subgrouping search space does not significantly alter relative classifier performance

We relaxed the parameters of our task enumeration heuristic to include subgroupings composed of up to three branches of at least five samples each which resulted in a larger total search space of 6483 subgroupings over the same 38 genes in METABRIC-(LumA). However, this did not uncover a large number of cases of divergent subgroupings that had not already been found using the original criteria (Additional file 8: Figure S7A–B). In cases such as SF3B1 the best available subgrouping had clearly already been identified given the limited number of mutation grouping combinations we could test given the modest size of the gene’s mutation landscape. The best subgroupings of TP53 and PIK3CA from this larger set converged even closer to their respective gene-wide counterparts, lending greater credence to the lack of a divergent set of mutations within these genes. MAP3K1 and CDH1 exhibited very modest improvements in classification performance using the enlarged pool of subgroupings: AUCs of 0.894 versus 0.889 and 0.823 versus 0.812 respectively for the best found subgroupings in the expanded and original search spaces respectively. However, neither of these subgroupings from the enlarged pool achieved cv-significance.

A notable exception was PTEN, in which a novel subgrouping consisting of terminating substitutions, missense mutations on the 5th exon, and frameshifts on the 7th exon was found to be cv-significant relative to the gene-wide task (AUC = 0.785 vs. AUC = 0.690). This is likely due to the dispersed landscape of PTEN mutations, with no single locus accounting for more than 5 out of the 51 PTEN point mutants in METABRIC-(LumA). Nevertheless, this was the only such case where a subgrouping discovered using the loosened enumeration criteria exhibited cv-significantly higher performance than the best-performing subgrouping found using the original criteria, and we hence deemed these gains as insufficient to justify a nine-fold increase in computational cost.

We also integrated copy number alterations (CNAs) into our task enumeration process. For every subgrouping used in the original experiment we created up to two additional classification tasks using the point mutations in the subgrouping alongside deep deletions or deep amplifications of the same gene in cases where these types of CNAs were present in at least five other cohort samples. However, this consistently failed to improve classification performance for cases such as GATA3, SF3B1, and AKT1 within which divergent subgroupings had already been found when not including CNAs in the set of mutations to predict (Additional file 9: Figure S8). In genes such as TP53 and MAP3K1 there were simply not enough deep CNAs present in the METABRIC-(LumA) cohort for us to test any subgroupings which included them. On the other hand, we found that using deep deletions alongside point mutations on the catalytic domain or on the C2 domain of PTEN led to a well-performing transcriptomic signature (50 samples; AUC = 0.750; cv-significant relative to using all PTEN point mutations). Including CNAs in our subgrouping enumeration also allowed us to better characterize genes such as ERBB2 (HER2) where deep amplifications on their own produced a cv-significantly superior expression signature (43 samples; AUC = 0.834) relative to using all point mutations of the gene (38 samples; AUC = 0.692) or all point mutations in conjunction with deep gains (80 samples; AUC = 0.731). Although most luminal A breast cancers exhibit HER2 negativity, a small number of HER2 positive breast cancers are classified as luminal A by PAM50 [63], and our results indicate that these samples are clearly distinguishable from the remainder of METABRIC-(LumA). While incorporating CNAs complemented what we discovered using solely point mutations, these findings were relatively few in number and we thus opted to focus on point mutation subgroupings for the remainder of our analyses.

### Breast cancer cohorts exhibit concordant divergence characteristics

**Fig. 2:**
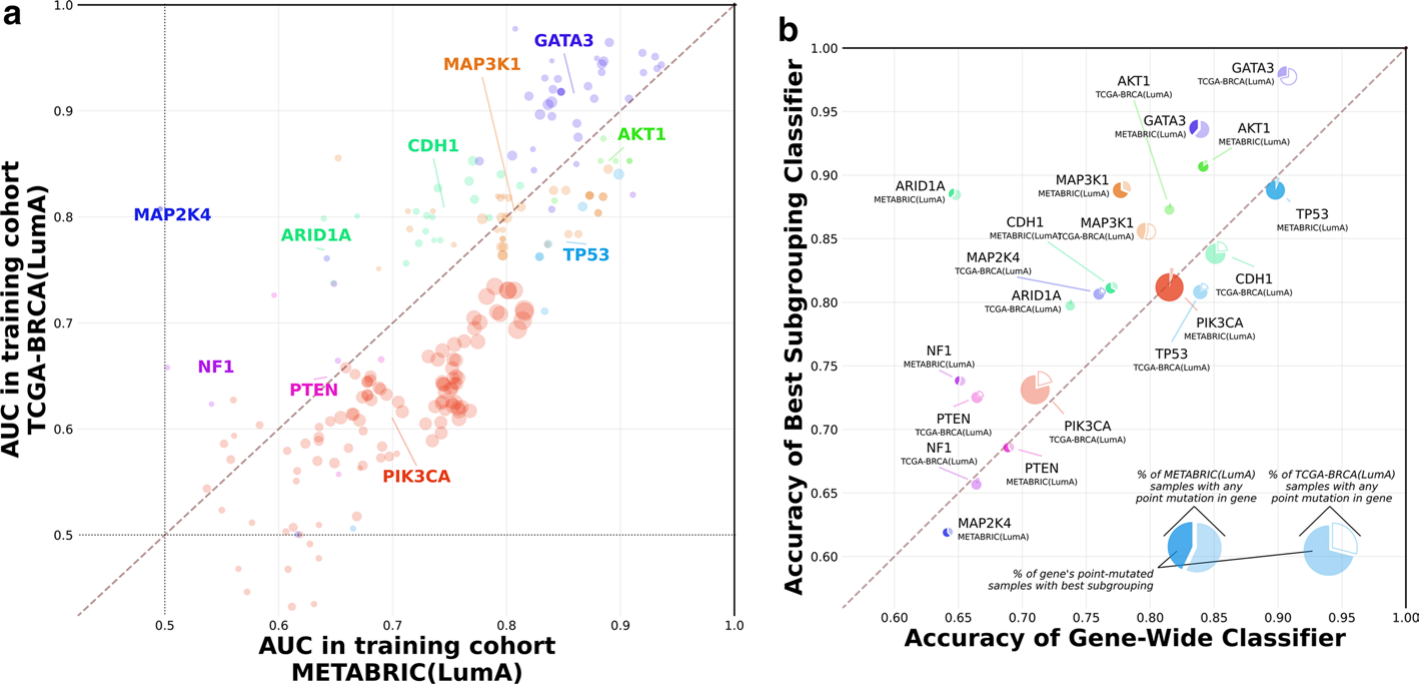
Subgrouping performance is consistent across breast cancer cohorts. Cancer gene subgrouping enumeration and classification was repeated using the luminal A sub-cohort of TCGA-BRCA. The colors for genes’ plotted points and pie charts correspond to those in Fig. 1. **a** Prediction AUCs for gene-wide classification tasks and subgrouping tasks enumerated in both METABRIC-(LumA) (x-axis) and TCGA-BRCA(LumA) (y-axis). Larger point size indicates a higher joint proportion of mutated samples (calculated as the geometric mean of the two cohort proportions). **b** Comparison of relative subgrouping performance (AUC) between cancer genes profiled in TCGA-BRCA(LumA) (filled-in pie charts) versus those profiled in METABRIC(LumA) (hollow pie charts)

We performed the same analyses on TCGA-BRCA [64] data to test whether the mutation grouping behavior observed in METABRIC generalizes to other breast cancer cohorts and is not simply an artefact of expression patterns specific to METABRIC or of overfitting within our classification tasks. The subgrouping enumeration procedure described above was repeated with the TGCA-BRCA luminal A sub-cohort consisting of 499 samples to identify 16 cancer genes containing 224 point mutation subgroupings of which 14 genes and 205 subgroupings had also been enumerated in METABRIC-(LumA). Training and evaluating classification tasks predicting the presence of these subgroupings using TCGA-BRCA(LumA) expression data revealed transcriptomic signature performance broadly concordant with results in METABRIC-(LumA), with a Spearman correlation of 0.796 across the AUCs for the 219 tasks tested in both cohorts (Fig. 2). This is despite the fact that expression calls in TCGA cohorts were made in an independent setting from METABRIC using next-generation sequencing profiling as opposed to microarrays.

This concordance persisted when comparing the relative performance of subgroupings within particular genes across the two cohorts. All four genes with at least ten tasks in both METABRIC-(LumA) and TCGA-BRCA(LumA) exhibited strong correlations between the two sets of AUCs (PIK3CA: 107 tasks, Spearman rho = 0.797; MAP3K1: n = 35, rho = 0.370; GATA3: n = 34, rho = 0.542; CDH1: n = 16, rho = 0.582). Of the six genes in METABRIC-(LumA) found to have divergent subgroupings according to cv-significance relative to the gene-wide task, three (GATA3, AKT1, MAP3K1) were also found to have divergent subgroupings by the same measure in TCGA-BRCA(LumA). Among the other three, SF3B1 had only 17 point mutants in TCGA-BRCA(LumA) and thus could not have any divergent subgroupings in TCGA-BRCA(LumA) in the first place, while ARID1A had 21 TCGA-BRCA(LumA) point mutants and only one enumerated subgrouping. The lack of divergent subgroupings in these cases can thus be explained by the relatively low incidence of their mutations and the smaller size of the TCGA-BRCA(LumA) cohort; it is also notable that the sole ARID1A subgrouping in this cohort did exhibit improved performance, albeit to a non-cv-significant extent (mutations not overlapping a SMART domain; n = 20; AUC = 0.798 vs. AUC = 0.738; $p_{Divg}$: 0.015). In the remaining case of CDH1, the nearly fourfold difference in point mutation incidence between the two cohorts (METABRIC-(LumA): 6.7%; TCGA-BRCA(LumA): 1.8%) suggests that variation in the composition of the cohorts or in the way mutations were called may be responsible for the lack of concordance in divergence.

To further validate the generalizability of subgrouping performance, we applied the models trained to predict subgroupings found in METABRIC-(LumA) to the TCGA-BRCA(LumA) cohort and vice versa. We found that these “transfer” classifiers were generally able to recapitulate both their absolute and their relative performances in the cohort that had been previously unseen to them. Across the 219 subgrouping tasks common to both cohorts, AUCs for tasks trained on METABRIC-(LumA) only dropped by an average of 0.038 when the models were applied to BRCA(LumA), and by an average of 0.00897 for models trained on BRCA(LumA) and applied to METABRIC-(LumA), with a Spearman correlation of 0.880 between the original and transfer AUCs in the former case and a Spearman correlation of 0.851 in the latter case. Furthermore, subgrouping classifiers for breast cancer genes such as GATA3 and MAP3K1 which had been found to be divergent from the corresponding gene-wide classifiers preserved their divergence in the transfer setting (Additional file 10: Figure S9).

The robustness of our findings was further underlined by obtaining comparable results when running the same experiment using TCGA-BRCA(LumA) expression calls produced by kallisto [65] as input rather than calls produced by RSEM [66] (Additional file 11: Figure S10A). Similar results were also obtained when using other combinations of the subtypes present in breast cancer instead of solely luminal A in both TCGA-BRCA and METABRIC (Additional file 11: Figure S10B-E). We thus conclude that the advantages of considering subgroupings within genes to model downstream transcriptomic effects are persistent when exposing these models to as yet unseen datasets, and that these mutation models generalize well across different breast cancer cohorts and expression quantification methods.

### Divergent cancer gene subgroupings are present across a variety of cancer types

**Fig. 3:**
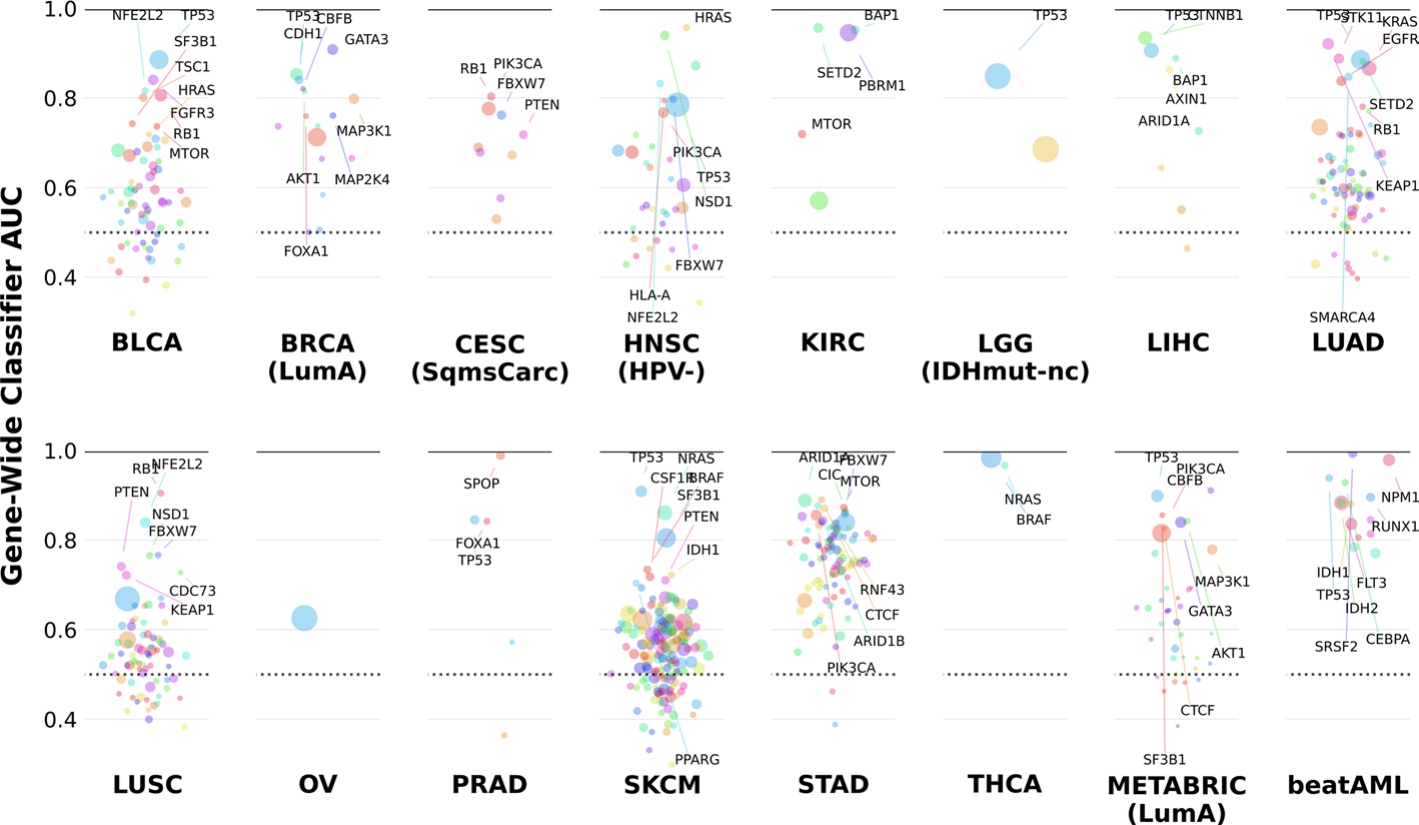
Many cancer genes’ point mutations have identifiable expression signatures. Our experiment attempted to predict the point mutations of a total of 200 cancer genes across 15 TCGA tumor cohorts as well as METABRIC and Beat AML using transcriptomic profiles. Shown are the AUCs for all 612 of these gene-wide tasks, with particularly well-performing classifiers highlighted. Point size corresponds to number of point-mutated samples in the given cohort

To further interrogate the presence of divergent alteration profiles across different tumor contexts, we repeated our enumeration and classification steps using the fourteen other cohorts in TCGA [64] with at least two hundred samples as well as the Beat AML cohort [67]. Three of these cohorts (TCGA-CESC(SquamousCarcinoma), TCGA-HNSC(HPV-), and TCGA-LGG(IDHmut-non-codel)) were formed by identifying molecular subtypes with unique transcriptomic profiles using unsupervised learning as was done for METABRIC-(LumA) and TCGA-BRCA(LumA). In total, 7097 subgrouping tasks spanning 200 different genes were completed across the 17 cohorts selected for training, in addition to 612 gene-wide tasks, 5163 CNA tasks, and 74,110 tasks constructed using randomly-chosen sets of samples (see Additional file 2: Tables S1 for a full list of tested subgroupings and for all subgrouping task AUCs). This revealed that gene-wide expression signatures can be trained for a number of cancer genes in most oncological contexts (Fig. 3). Of the 612 gene-wide tasks, 462 had at least one enumerated point mutant subgrouping for the same cohort and gene, and in 106 cases at least one of these subgroupings’ AUC was cv-significantly higher than that of the gene-wide task, with 57 of these divergent subgrouping tasks achieving an AUC of at least 0.7.

Divergent subgroupings are a feature of not just breast cancer but of many other tumor types as well (Additional file 12: Figure S11). For example, within the context of prostate adenocarcinoma (TCGA-PRAD), mutations of FOXA1 that overlap with the fork head binding domain are much easier to predict than all FOXA1 point mutations taken together (22/31 FOXA1 mutants; AUC = 0.953 vs. AUC = 0.842; cvSig; $p_{Divg}$: 0.033). This is consistent with the importance of such domains in guiding the regulatory functions of transcription factors as well as with previous characterizations of the functional divergences present within FOXA1 mutations in prostate cancer cohorts [68–70]. In the HPV-subtype of head and neck squamous carcinomas (TCGA-HNSC), NSD1 was found to have a divergent subgrouping consisting of frameshifts and nonsense mutations (37/52 samples; AUC = 0.977 vs. AUC = 0.940; cvSig; $p_{Divg}$: 0.95). Thus we can deduce that the fifteen missense mutations of NSD1 present in the cohort have a much weaker downstream effect, especially as the same subgrouping with nonsense mutations replaced by missense mutations had relatively poor classification performance (34/52 samples, AUC = 0.822, $p_{Divg}$: 0.99). EGFR contains two major hotspots (E746-A750del and L858R) in the lung adenocarcinoma cohort (TCGA-LUAD) which together accounted for 37 of the samples in the best found subgrouping (48/73 samples, AUC = 0.934 vs. AUC = 0.838, cvSig; $p_{Divg}$: 0.029). In conjunction with the considerably lower performance of the best classifier containing only one of the two hotspots (21/73 samples, AUC = 0.913, $p_{Divg}$: 0.65) this implies that these two loci have similar or at the very least highly complementary impacts on the transcriptome.

In the case of KRAS, although we were unable to find any subgroupings that were divergent according to classification performance in neither TCGA-LUAD nor TCGA-STAD, we did discover that in TCGA-LUAD the subgrouping composed of the G12V and G12D substitutions (55/153 KRAS point mutants; AUC = 0.76; $p_{Divg}$: 0.961) exhibited a divergent coefficient profile from KRAS subgroupings containing the dominant G12C hotspot (Additional file 4: Figure S3). This is concordant with previous work demonstrating G12D mutants require targeted interventions distinct from those directed at G12C mutants [27].

Examining the subgrouping task characteristics of genes frequently mutated in multiple cohorts allowed us to make inferences about the sensitivity of these mutations’ transcriptomic effects to the tumor contexts in which they occur. For instance, we can produce a well-performing signature for TP53 variants in multiple cancer cohorts including melanoma (67 mutated samples in TCGA-SKCM; AUC = 0.909), bladder cancer (200 muts in TCGA-BLCA; AUC = 0.886), and lung adenocarcinoma (264 muts in TCGA-LUAD; AUC = 0.885) in addition to the signature found in luminal A breast cancer already described above. However, TP53 mutations do not exhibit cv-significant divergence in any of these cancers, nor in any of the other ten cohorts used in this study in which its subgroupings were enumerated (Additional file 13: Figure S12). In addition to supplying more evidence for the homogeneity of the TP53 mutation landscape, this also suggests that the effects of TP53 mutations are consistent across different disease contexts.

To confirm this, the transfer validation method that was used to compare the performance of trained models between our two breast cancer cohorts was extended across all of the cohorts we used for training. Transferring trained TP53 classifiers across disease contexts revealed that its transcriptomic signature generally performs well even when it is applied to a novel tumor context, reflecting not only the robustness of our classification pipelines but also the ubiquitous nature of the downstream effects associated with TP53 point mutations (Additional file 14: Figure S13). On the other hand, PIK3CA exhibits divergence in some tumor contexts such as stomach cancer and bladder cancer but not in most others, suggesting that recurrent PIK3CA mutations may result in unique downstream transcriptional signals depending on the unique cancer context in which they developed (Additional file 13: Figure S12). This conclusion was further supported by the poor transfer performance of PIK3CA models (Additional file 14: Figure S13).

In contrast to this, NFE2L2 is associated with both a robust downstream signature and at least some divergence in all three cohorts in which its subgroupings were enumerated, with the subgrouping consisting of mutations on the 2nd exon featuring as the most divergent in each case (TGCA-BLCA: 20/27 muts, AUC = 0.927, $p_{Divg}$: 0.265; TCGA-LUSC: 67/75 muts, AUC = 0.897, $p_{Divg}$: 0.289; TCGA-HNSC(HPV-): 21/31 muts, AUC = 0.915, $p_{Divg}$: 0.0176) (Additional file 13: Figure S12). Although this subgrouping only achieved cv-significance relative to the gene-wide task in TCGA-BLCA and TCGA-LUSC, in TCGA-HNSC(HPV-) it clearly approached cv-significance, with nine of the ten cv AUC comparisons showing the subgrouping outperforming the gene-wide task. Furthermore, its subgrouping models performed well in transfer validation and continued to outperform models trained using all of the gene’s point mutations in transfer contexts (Additional file 15: Figure S14). These subgrouping models are therefore especially likely to preserve their performance when applied to novel cohorts or patient samples, which is especially important in a variety of clinical settings where they would be implemented.

### Subgroupings outperform mutation subsets chosen using variant significance metrics

We have already compared the classification performance with our mutation subgroupings against the performance when using all point mutations for the corresponding gene, as well as against the performance when using sets of samples chosen at random from both the training cohort as a whole and the set of samples carrying any point mutation on the gene. To further validate our approach, we compared the performance of classifiers tasked with predicting the presence of our mutation subgroupings against those predicting subsets of mutations constructed using existing metrics designed to capture the impact of mutations on cancer processes. For each gene with enumerated subgroupings in a cohort we thus created a classification task for each possible threshold value of the PolyPhen and SIFT scores [71, 72] assigned to its variants that resulted in a unique set of at least 20 samples carrying a mutation of the gene satisfying the threshold. This allowed us to evaluate the relative efficacy of the transcriptomic signature trained using a subgrouping containing *n* mutated samples of a gene against that of a signature trained using a subgrouping containing the top *n* mutants according to PolyPhen or SIFT wherever these scores were available.

Out of a total of 416 mutated genes in cohorts for which PolyPhen and SIFT data was available and in which any subgroupings were enumerated, 97 had at least one subgrouping cv-significantly divergent relative to the gene-wide task, but only 35 had at least one of these threshold-based subgroupings passing the same test of significance, of which 22 also belonged to the cases with at least one cv-significant subgrouping. In genes such as EGFR in TCGA-LUAD and NFE2L2 in TCGA-HNSC(HPV-) our method of discovering subsets of mutations outperformed any possible choice of cutoff of the above metrics (Additional file 16: Figure S15). Using PolyPhen and SIFT cutoffs also failed to find divergence within genes such as TP53 and PIK3CA where we had not discovered any divergent subgroupings (Additional file 16: Figure S15) further suggesting that divergences within the mutation profiles of these genes, if they do exist, are overshadowed by a common expression program. Our subgrouping enumeration method can thus outperform other approaches for evaluating the potential impact of oncogenic mutations, and highlights the importance of incorporating a variety of biological priors when characterizing the relationships between mutations and their tumorigenic effects.

### Subgrouping classifier output reveals the structure of downstream effects within cancer genes

Comparing the performances of transcriptomic signatures for different subsets of mutations within cancer genes has allowed us to identify divergences within them. However, this analysis does not on its own pinpoint the nature of the differences within these genes’ mutations that are responsible for this observed heterogeneity—a subgrouping could have a transcriptomic profile that diverges from that of its parent gene for a variety of reasons. For instance, it is possible that the mutations of the gene not belonging to the subgrouping are functionally silent. Another possibility is the existence of multiple transcriptomic programs within the gene that are complementary or orthogonal to one another, each of which can be uniquely mapped to a subset of the gene’s mutations. We thus investigated the output of the signatures we trained for these subgroupings to better understand the mechanisms driving the downstream transcriptomic effects of tumorigenic alterations.

For subgroupings that had been identified as divergent in the cohorts we included in our experiment, we examined the mutation scores their expression classifiers returned for the mutations on the same gene not belonging to the subgrouping (Additional file 17: Figure S16). This helped us to characterize the relationships that were responsible for the observed divergence between each subgrouping and the remaining mutations on the gene in which they were found. For example, we were able to confirm that mutations falling outside of the best found subgroupings of both AKT1 and SF3B1 in METABRIC-(LumA) behave like wild-type samples according to our classifiers’ scores, which is consistent with the subgroupings partitioning these genes’ mutations into active and inactive subsets. Similar behavior was observed especially in cases such as BRAF in TCGA-SKCM and TGCA-THCA in which the mutational landscape is composed of a dominant hotspot and a collection of other seemingly inactive variants. On the other hand, in cases such as TP53 and PIK3CA the mutations not belonging to the best found subgrouping were nevertheless consistently mistaken by the classifier as belonging to the subgrouping, lending further weight to the hypothesis that these genes’ mutational landscapes are relatively homogeneous. These results have immediate clinical utility when evaluating a variant of unknown significance in a cancer context.

This approach also helped to explain why synonymous mutations were included in the best found subgrouping of NF1 in METABRIC-(LumA). Although one should expect that these variants would have downstream effects equivalent to that of NF1 wild-types when compared to other types of NF1 point mutations, we found that a classifier trained to predict NF1 nonsense and synonymous mutations performed significantly better than the NF1 gene-wide classifier (21/48 NF1 point mutants, AUC = 0.782 vs. AUC = 0.660, down-sampled confidence = 0.97) as well as the classifier trained to predict NF1 nonsense and missense mutations (31/48 NF1 point mutants, AUC = 0.614). Because there were fewer than 20 samples total bearing NF1 nonsense mutations, no classifier was trained on them in isolation, which was also true of synonymous mutations.

Examining the distributions of the scores returned by these classifiers revealed that the combined NF1 nonsense and synonymous mutation subgrouping’s classifier was not only better able to distinguish between its own mutations and the remaining samples in the cohort, but it also did a better job of separating other NF1 mutations from NF1 wild-types than the gene-wide classifier, and especially NF1 missense mutations (Additional files 18: Figure S17A). Furthermore, it successfully predicted the presence of synonymous mutations in held out samples. We thus conclude that synonymous NF1 variants are very likely to have downstream transcriptomic effects aligned with those of active NF1 mutations. Although this finding contradicts the intuition that such mutations should not imbue significant downstream impacts, it is less surprising in light of prior work demonstrating that these mutations can indeed enact non-trivial effects on splicing, transcript folding/stability, translational rates, co-translational folding/stability, and degradation [73, 74]. Although NF1 splice mutations are often mistaken for silent mutations by sequencing methods [75, 76], evidence that synonymous mutations of the NF1 gene are selected in cancers such as T-cell acute lymphoblastic leukemia [77] signals a need for more research in this area.

**Fig. 4:**
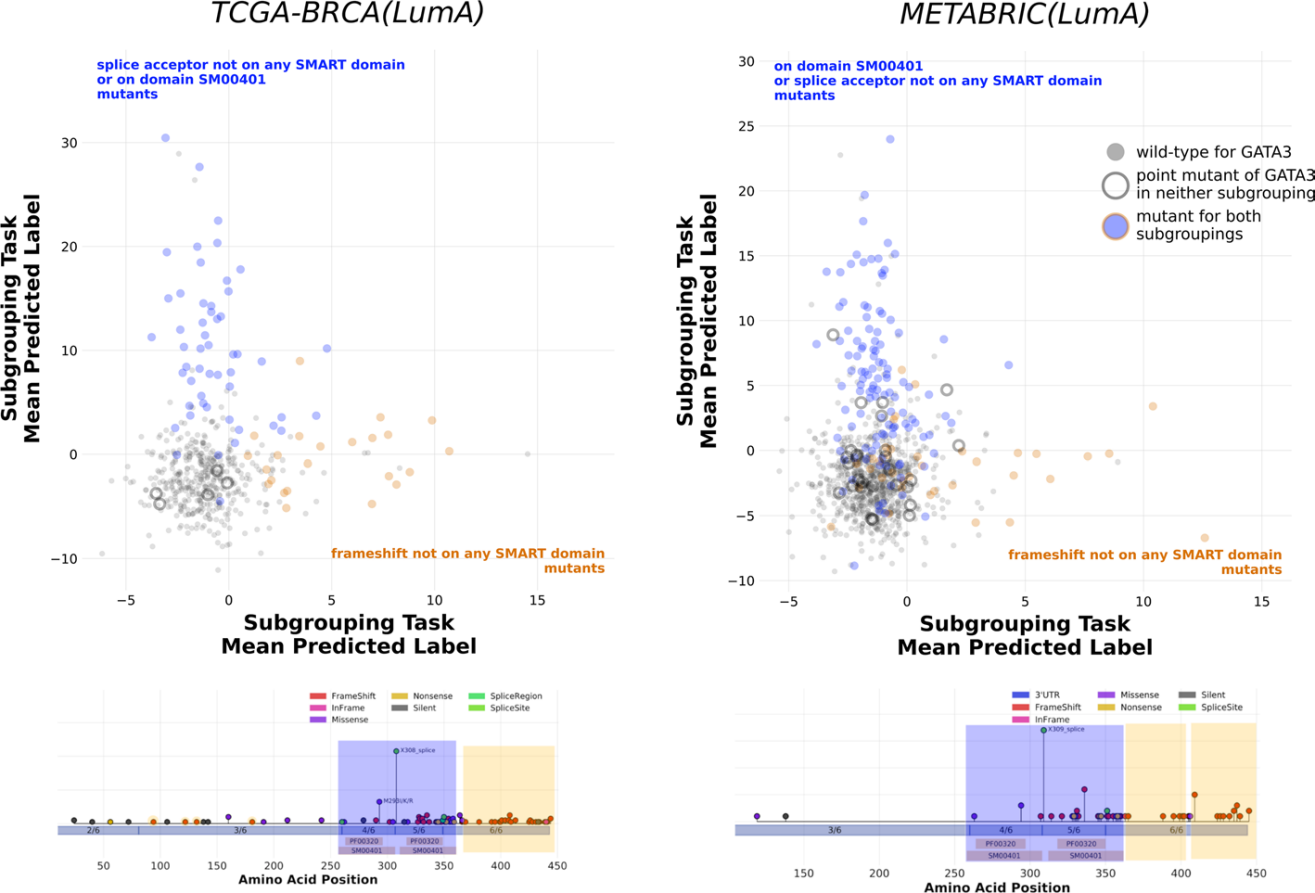
GATA3 downstream effects can be decomposed into two orthogonal axes. Amongst the divergent subgroupings enumerated for GATA3 in our breast cancer cohorts, we found a pair of non-overlapping subgroupings that produced mutation scores with no correlation with one another in both METABRIC(LumA) and TCGA-BRCA(LumA). Each cohort sample is represented by a point, with samples shaded according to whether they carried a mutation in one of the subgroupings, neither, or in both as indicated by the figure labels and legend

An altogether different type of pattern was discovered within GATA3 in breast cancer where we found that GATA3 mutations not included in the best found subgroupings tended to have predicted scores between those assigned to samples carrying the subgrouping and GATA3 wild-types (Additional file 18: Figure S17B–D). Further examination revealed that GATA3 mutations can be decomposed into disjoint pairs of subgroupings corresponding to whether they overlap with the zinc finger domain or the X308 splice site hotspot whose predicted scores were orthogonal to one another. This behavior was present in both METABRIC(LumA) and TCGA-BRCA(LumA), thus revealing the presence of two independent expression programs within GATA3 that are consistent across different breast cancer cohorts (Fig. 4). This builds upon existing research demonstrating that GATA3 mutations can be partitioned into subsets with different functions and clinical outcomes by providing a transcriptomic characterization of these groupings [38, 78].

### Subgroupings enrich the characterization of drug response in cell lines

Do these divergences in downstream effects lead to divergent responses to pharmacological treatments? To answer that question, we tested the performance of our subgrouping classifiers in predicting response to drug interventions in cancer cell lines. We transferred the classifiers we trained in each of our cohorts to the CCLE cohort [79], which contains -omic and drug response data for 990 cell lines. For each classification task, we calculated the correlation between the mutation scores predicted for the CCLE cohort and drug response as measured by AUC50 for the 265 drugs which had response profiles available in at least 100 of the cell lines in the cohort where expression calls had also been made. We thus found that many subgroupings which exhibited divergent classification performance in the training cohort also yielded divergent associations with these clinical phenotypes (Additional file 19: Figures S18).

**Fig. 5:**
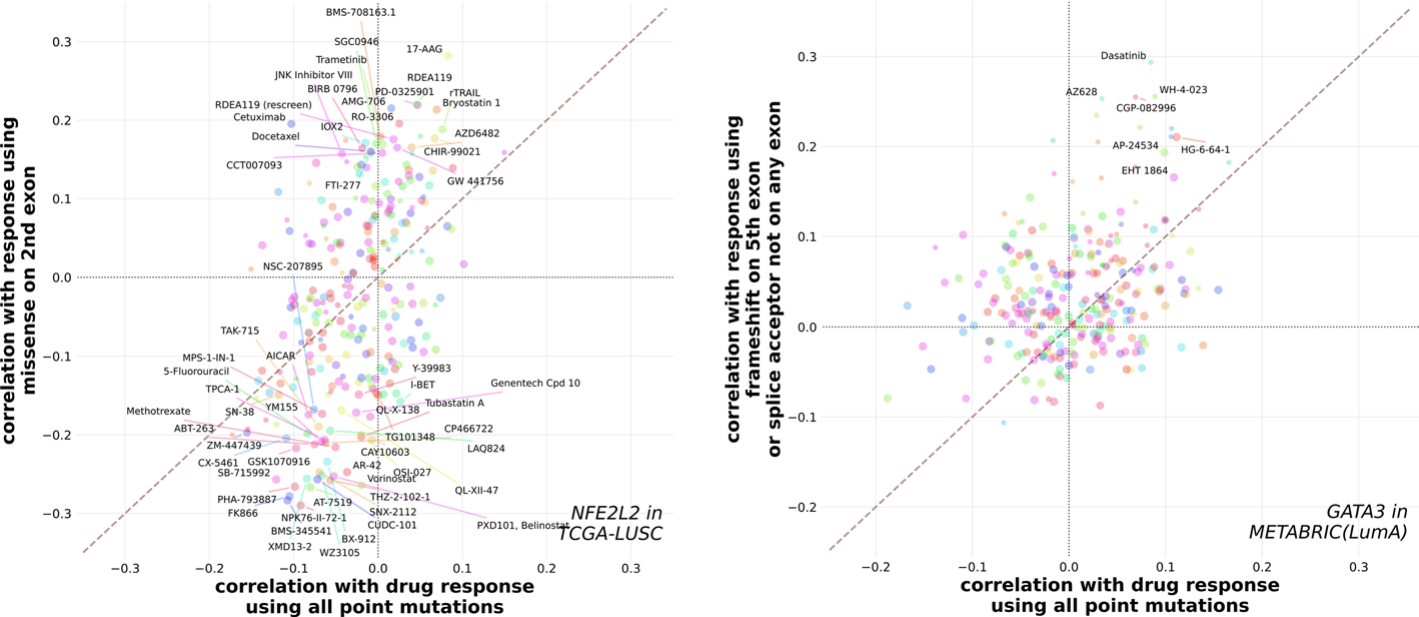
Using subgroupings improves concordance with clinically relevant phenotypes. We applied our trained classifiers to the CCLE cohort and computed the Spearman correlations between the scores returned by the classifiers and drug response for 265 compounds with AUC50s measured in at least 100 cell lines which also had expression calls available. For NFE2L2 in TCGA-LUSC and GATA3 in METABRIC(LumA) we compared these correlations for the gene-wide classifier and the classifier of the best found subgrouping. Points correspond to individual drugs, with the area of each point proportional to the number of cell lines for which AUC50s were available for the given drug. Correlations were multiplied by $-1$, and thus higher correlations correspond to stronger association with increased sensitivity of the cell lines to the compound in question. Labels have been added for drugs with Spearman rank-order test *p* values of less than 0.001 for the subgrouping correlation but greater than 0.001 for the gene-wide correlation

For example, we compared the correlations between drug sensitivity and the scores produced by the NFE2L2 gene-wide classifier to those of the scores given by NFE2L2’s best found subgrouping classifier (missense mutations on the 2nd exon) (Fig. 5). NFE2L2 encodes NRF2, a major cytoprotective regulator of the antioxidant response to intrinsic and extrinsic stressors. While NRF2 activation is protective in diseases of inflammation, in the context of cancer NRF2 promotes oncogenic signaling, growth, and survival [80–82]. While the NFE2L2 gene-wide classifier scored cell lines in a way that correlated similarly across the majority of the drugs assessed, the scores assigned by the subgrouping classifier were better able to associate or disassociate samples with vulnerabilities to various drug interventions. The highest positive correlation between a classifier’s scores and sensitivity to a drug was the subgrouping classifier’s association with 17-AAG. 17-AAG is an HSP90 inhibitor and geldanamycin derivative that displays synthetic lethality with aberrant NRF2 activity [83]. Several target genes of NRF2 metabolize geldanamycin derivatives into more potent HSP90 inhibitors, which selectively enhances toxicity in NRF2-overexpressing cells [83]. Exon 2 includes Neh2 domain that is responsible for binding to KEAP1, a key regulator of NRF2. This finding provides strong biological grounding for why a classifier trained to predict missense mutations in exon 2 of NFE2L2 might also be able to predict sensitivity to a drug like 17-AAG.

We also looked at drug sensitivity between divergent subgroupings of GATA3 mutations. The scores returned by the best GATA3 subgrouping in METABRIC-(LumA) consisting of mutations on the 5th exon and splice site mutations not on any exon consistently had stronger correlations with increased sensitivity to a wide range of drugs interrogated in CCLE, including tyrosine kinase inhibitors such as Dasatinib and Lapatinib (Fig. 5). Dasatinib is a selective inhibitor of SRC-family kinases [84–87]. Zinc finger 2 (ZnFn2) mutations occur in the 5th exon of the GATA3 gene, and typically truncate the C-terminus. These mutations decrease canonical GATA3 binding, and are generally characterized as loss of function [78], however it was recently discovered that Znfn2 mutant GATA3 can localize to a novel suite of target genes and exhibit transcriptional reprogramming in favor of epithelial to mesenchymal transition (EMT) [38]. SRC and SRC-family kinases are also known to regulate EMT in solid tumors [88, 89], thus providing an indirect link that may explain observed Dasatinib sensitivity of cells with strong GATA3 Znfn2 signal. These findings indicate that transcriptionally divergent subgroupings can help us better characterize the impact of mutations on processes that may influence the efficacy of pharmacological interventions.

## Discussion

Although genomic heterogeneity is an intrinsic property of tumor cohorts [1, 19, 20], our limited understanding of the downstream consequences of this heterogeneity remains a major obstacle to furthering the efficacy of treatments in precision oncology [7, 9–11]. To address this issue, we have introduced a framework for exploring and characterizing the diversity of alteration landscapes in genes frequently mutated in several cancers. This approach leverages biological priors about the structure of variants recurrent in cancer along with a modern computing cluster’s ability to train and test expression signatures for thousands of variant groupings in parallel.

In addition to ascertaining robust gene-wide transcriptomic signatures in many cases, this approach allowed us to systematically identify cancer genes containing subsets of mutations with functional effects that diverge from the remaining mutations. Considering subgroupings of mutations allowed us to find expression profiles associated with a gene implicated in tumorigenesis in many cases where it was otherwise difficult or impossible, and to discern which mutations in a gene are particularly likely to have a quantifiable downstream effect. The gains from considering a variety of mutation subgrouping tasks were far greater than from using more sophisticated classification algorithms, and often yielded more accurate models than using other methods to identify significant variants. These results are striking in that predicting the presence of a rarer type of mutation should, everything else being equal, be more difficult owing to decreased statistical power. Subgrouping signatures also exhibited strong performance in tumor cohorts to which they were not exposed during training, as well as improved association with drug response in cell lines.

In cohorts such as TCGA-OV our framework struggled to find any well-performing signatures as there was a paucity of mutations present in at least twenty samples, with all 109 enumerated subgroupings representing mutations of TP53. This reflects the reliance of our approach on frequently recurring mutations; given the limited size of the cohorts publicly available at this time, our framework is bound to be most effective when applied to dominant driver mutations. Nevertheless, these mutations have an outsized role in cancer processes, and our approach is still able to shed light on the characteristics of downstream effects of these important players in tumorigenesis.

## Conclusions

Our findings confirm that genes with divergent mutation profiles are ubiquitous in cancer. Furthermore, they demonstrate that no characterization of the downstream effects of genes implicated in tumorigenesis is complete without taking these divergences into account. This builds upon previous studies showing that machine learning models trained on transcriptomic data can be used to characterize particular variants implicated in tumorigenesis [13–18] by expanding this methodology across a comprehensive catalogue of variant subgroupings within cancer genes.

Our exploration of the subgrouping search space allowed us to construct more robust models linking the genome and transcriptome in tumor cohorts, as well as to predict the effects of mutations of unknown significance and to characterize the relationships between different downstream effect axes extant within genes active in cancer processes. The detection of divergent alteration subgroupings has the potential to improve the specificity of precision treatments, aid in patient stratification, and to anticipate otherwise unexpected and undesirable therapeutic outcomes. Furthermore, discovering subgroupings composed of mutations with convergent downstream effects may guide efforts to reposition existing pharmaceutical interventions to orthogonal scenarios that resemble approved clinical indications. This approach thus allows us to construct a more complete compendium of expression signatures associated with driver events in cancer, and illustrates that identifying subsets of mutations with unique transcriptomic signatures can yield robust and actionable biological insights.

## Methods

### Cohort data preparation

We used a total of 18 publicly available tumor cohorts in this study, which included 15 individual cohorts from TCGA as well as METABRIC, Beat AML, and CCLE [31, 64, 67, 79]. These cohorts were selected on the basis of the availability of all three of expression, variant, and copy number data for the samples they contained (except for Beat AML, for which CNA calls were not made), as well as sufficient size (at least 200 samples with all three data types collected). Cohorts such as TCGA-COADREAD and TCGA-GBMLGG which are agglomerations of other cohorts were omitted.

For TCGA cohorts, Illumina RNAseq RSEM-normalized expression calls and GISTIC2.0 copy number calls were downloaded from the Broad Firehose portal [90], while TCGA variant calls were downloaded from the Synapse portal for the MC3 pan-cancer analysis pipeline [91, 92]. Expression, copy number, and variant data for the METABRIC and CCLE datasets were downloaded from cBioPortal [93–95].

We applied UMAP (version 0.3.10) to project the expression profiles of the samples in each cohort into a two-dimensional space for easier interrogation of the global structures present within the transcriptome [32]. These projections were compared against annotations of known molecular subtypes in cohorts where such annotations were available. We created sub-cohorts where UMAP transcriptome clusters overlapped with these subtypes (see Additional file 1: Figures S1). Cases such as TCGA-SARC which initially passed the 200-sample threshold but had to be divided into sub-cohorts that did not meet the threshold were omitted from further analysis. Molecular subtype annotations for TCGA cohorts were provided by the Korkut Lab as part of the PCAWG Consortium; for METABRIC these annotations were downloaded from cBioPortal.

### Defining mutation subgroupings

Our mutation subgrouping method is based on organizing the genomic alterations present in a cohort according to various properties that mutations can have in common. The particular properties used in this study are were based on the fields available as output through the Ensembl Variant Effect Predictor (VEP v99.2) which was used to process the variant calls of each cohort in order to produce uniform mutation data across all datasets [96]. These properties were:

- **Exon** The exon on which the mutation is located. The value ‘.’ was given to mutations such as splice site deletions which are not assigned to a specific exon.
  *e.g.* Exon = 5, Exon = 2
- **Amino Acid Location** The amino acid or acids affected by the mutation. The value ‘.’ was given to mutations for which this property is not applicable, such as intronic mutations.
  *e.g.* Location = 1047, Location = 274
- **Amino Acid Substitution** The specific protein substitution that takes place as a result of the mutation.
  *e.g.* H1047R, V600E
- **Consequence** The functional consequence of the mutation.
  *e.g.* missense, stop gain, frameshift
- **SMART Domain** The SMART protein domain on which the mutation rests. Can also take on the value “no overlapping domain”.
  e.g. SM00233
- **Pfam Domain** The same as above but with Pfam protein domains.
  e.g. PF00853

A subgrouping is thus defined by a nested combination of values chosen for one or more of these attributes. For example, a single hotspot mutation in PIK3CA can be represented by the subgrouping $\{ AAsub=H1047R \}$. We can define the same subgrouping using additional properties: $\{ Exon=21{:}\,AAloc=1047{:}\,AAsub=H1047R \}$. These additional properties are redundant in this case, as naturally all H1047R substitutions are located at amino acid 1047 and in turn all of the alterations at this amino acid are located on the 21st exon of PIK3CA. Nevertheless, we can expand this subgrouping to include other PIK3CA mutations which may or may not be functionally similar to H1047R. Thus we could consider the subgrouping $\{ Exon=21{:}\,AAloc=1047{:}\,AAsub=(H1047R~ or~ H1047L) \}$ to test the hypothesis that the particular amino acid that replaces the wild-type at this hotspot does not have an impact on downstream effects. Likewise, the subgroupings $\{ Exon=21{:}\,(AAloc=1047{:}\,AAsub=H1047R)~ or~ (AAloc=1049{:}\,AAsub=G1049R) \}$ and $\{ Exon=10{:}\,AAloc=542{:}\,AAsub=E542K ~ or~ Exon=21{:}\,AAloc=1047{:}\,AAsub=H1047R \}$ can be used to compare hotspots at different loci within PIK3CA. We can also choose other properties to construct the same subgrouping based on which attributes of PIK3CA alterations we believe to be the most important in determining downstream effects: $\{ Consequence=missense{:}\,AAsub=H1047R \}$, $\{ Domain=SM00146{:}\,AAsub=H1047R \}$, and so on.

Although these hierarchies allow for a fairly extensive search over the possible subsets of the mutations of a gene occurring in a cohort of samples, they do not offer a firm lower bound for finding the maximally divergent subgrouping. For our purposes, however, it is sufficient to detect at least one statistically significant divergent partitioning. Since we are systematically scanning all frequently altered genes across many cohorts, computational cost and statistical loss due to multiple hypothesis testing are limiting constraints. We found that our sampling heuristic based on biological priors can still elucidate multiple interpretable divergent subsets while pruning the search space down to a manageable size.

### Enumeration of classification tasks in tumor cohorts

Cancer genes were identified using the OncoKB repository, with only genes included in at least one of the “Vogelstein”, “SANGER CGC(05/30/2017)”, “FOUNDATION ONE”, and “MSK-IMPACT” lists being included for further analysis [97]. In each cohort we considered the grouping of all point mutations in each such gene (referred to as the gene-wide task) and also sought to generate subgroupings of mutations within these genes.

We pruned the subgrouping search space by only using the four ordered mutation property hierarchies listed below, with the reasoning that a sizeable proportion of biologically relevant subgroupings of mutations could be generated using one of these combinations:

- $$\texttt {Exon} \rightarrow \texttt {AA Location} \rightarrow \texttt {AA Substitution}$$
- $$\texttt {Consequence} \rightarrow \texttt {Exon}$$
- $$\texttt {SMART Domain} \rightarrow \texttt {Consequence}$$
- $$\texttt {Pfam Domain} \rightarrow \texttt {Consequence}$$

To further prune our search space, we only used subgroupings corresponding to a single branch containing at least twenty samples in one of these hierarchies as well as subgroupings corresponding to two branches each with at least ten samples. Branches did not have to terminate at a leaf node of the hierarchy. For example, using the combination $\texttt {Consequence} \rightarrow \texttt {Exon}$, we could test $\{PIK3CA{:}\,missense\}$ as well as $\{PIK3CA{:}\,missense{:}\,Exon=10\}$, $\{PIK3CA{:}\,missense{:}\,Exon=21\}$, and $\{PIK3CA{:}\,missense{:}\,Exon=21~ or~ PIK3CA{:}\,Silent\}$, but not $\{PIK3CA{:}\,missense{:}\,Exon=(10~ or~ 21)~ or~ PIK3CA{:}\,synonymous~ or~ PIK3CA{:}\,stopgain\}$.

To test the marginal benefit of relaxing this requirement, we also tested three-branch subgroupings with at least five samples in each branch and twenty samples in total in the case of METABRIC-(LumA). In all cases, subgroupings that contained all of the mutations of a gene in a cohort were discarded as being equivalent to the gene-wide task, which occurred in cases where the mutation hierarchy contained no more than two branches in total for a particular gene.

### Construction of classification tasks

A classification task was created for each of these enumerated subgroupings in a given cohort. To obtain a background distribution of predictive performance, we also added classification tasks using sets of samples randomly chosen from the cohort. Ten such sets were created for each subgrouping already found, each of which contained the same number of samples as the number of samples carrying a mutation in the subgrouping in question. Five of these “random” subgroupings for each actual subgrouping chose samples from the entire cohort, while the other five only chose from the set of samples containing any point mutation in the gene mutated for the subgrouping.

Further classification tasks were added by considering copy number alterations as identified using discretized GISTIC 2.0 calls. For each of the non-random subgroupings described above, we created two new subgroupings by adding the set of samples carrying deep amplifications ($+$ 2) in the same gene as well as the set carrying deep deletions (− 2). In cases where the given gene did not have at least five samples carrying the CNA to be added to the subgrouping, the corresponding subgrouping was excluded from further consideration. In genes where there were at least twenty deep amplifications or twenty deep deletions, we created a classification task containing just these CNAs of the gene.

Classification tasks were also constructed by dynamically discretizing PolyPhen and SIFT scores wherever these scores were available for the cohort (i.e. in TCGA cohorts) [71, 72]. For each combination of mutated gene and variant significance metric, we enumerated all possible thresholds of the metric observed over variants of the gene in a cohort that yielded a unique subgrouping with at least twenty samples harboring a mutation in the gene satisfying the threshold value (in the positive direction in the case of PolyPhen and the negative direction in the case of SIFT). For example, for AKT1 in TCGA-BRCA(LumA), we found the PolyPhen subgroupings $>=0.006$ and $>=0.999$ (and no SIFT subgroupings), while for TP53 in TCGA-STAD we found 29 PolyPhen subgroupings ($>=0.002,>=0.09,>=0.275,\cdots ,>=1.0$) and 12 SIFT subgroupings ($<=0.8,<=0.13,<=0.11,\cdots ,<=0$).

### Training and evaluation of classifiers to identify transcriptomic signatures associated with subgroupings

Expression and variant data in each cohort was filtered to only include protein-coding genes on non-sex chromosomes prior to classifier training. Genes whose expression was missing in any of our cohorts were removed. Remaining expression data was then filtered to exclude gene features in the bottom decile according to average value across the cohort before being log-normalized and then scaled using z-scores for each genetic feature. In each task we further excluded expression features associated with genes on the same chromosome as the gene containing the task’s subgrouping.

Each classification task consisted of predicting a vector of binary mutation labels using this processed expression matrix for a given cohort. The label for each sample in a task was ‘True‘ if and only if it harbored any mutation within the subgrouping, or if it was randomly chosen from the set of cohort samples or the set of gene mutants as applicable for random background subgroupings. Predictions were made using the following algorithms implemented in scikit-learn (version 0.21.3), with any parameters not explicitly listed above being set to the default value:

- **Logistic Ridge Regression**
  sklearn.linear_model.LogisticRegression
  - tuning over eight values of *C*: $[10^{-7}, 10^{-6}, 10^{-5},\ldots ,10^{0}]$
  - solver = ‘liblinear’, penalty = ‘l2’, max_iter = 200, class_weight = ‘balanced’
- **Support Vector Machine**
  sklearn.svm.SVC
  - tuning over eight values of *C*: $[10^{-3},10^{-2},10^{-1},\ldots ,10^{4}]$
  - kernel = ‘rbf’, gamma = ‘scale’, probability = True, cache_size = 500, class_weight = ‘balanced’
- **Random Forests**
  sklearn.ensemble.RandomForestClassifier
  - tuning over eight values of $min\_samples\_leaf$: [1, 2, 3, 4, 6, 8, 10, 15]
  - n_estimators = 5000, class_weight = ‘balanced’
- **Logistic Ridge Regression (deeper tuning)**
  sklearn.linear_model.LogisticRegression
  - tuning over $$C=[10^{-8.2}, 10^{-7.8}, 10^{-7.4},\ldots ,10^{-4.2}]$$
  - solver = ‘liblinear’, penalty = ‘l2’, max_iter = 200, class_weight = ‘balanced’

Forty classifiers were fit for each task corresponding to ten iterations of fourfold cross-validation. The samples in each cohort were partitioned into quarters at random ten times; each classifier was thus tuned and trained on three such quarters before being asked to make predictions on the remaining quarter of samples. The same forty training and testing sub-cohorts were used across all tasks on a given cohort. Task classifiers was tuned by training the classifier using each of the values in the classifier’s hyper-parameter tuning grid on four randomly-chosen subsets consisting of 80% of the training sub-cohort. The accuracy for each hyper-parameter tuning value was measured by taking the worst AUC across its four trained classifiers on the remaining 20% of the samples in the training sub-cohort. The best hyper-parameter value according to this metric was then used when training the classifier on the entire training sub-cohort before applying it to the entire testing sub-cohort.

Classifier task performance on these testing sub-cohorts was measured using AUC as calculated by averaging predicted mutation scores for each cohort sample from all ten cross-validation iterations, segregating scores for mutated samples and wild-type samples, and then calculating the probability that a randomly-chosen mean score for a mutated sample was greater than a randomly-chosen mean score for a wild-type sample across all possible such sample pairs. Likewise, cv-AUCs were calculated for each cross-validation iteration separately by using that iteration’s scores for mutated and wild-type cohort samples. Classifier task performance was further measured on each of the cohorts other than the one the classifier was trained on by applying each of the forty trained classifier iterations to their processed expression data. AUCs for these “transfer” experiments were calculated using the same sample-average method as described in the within-cohort case, this time with forty classifier output values for each sample.

For two subgrouping tasks *X* and *Y* we used the corresponding cv-AUCs $\{x_1, x_2, \ldots , x_{10}\}$ and $\{y_1, y_2, \ldots , y_{10}\}$ to create a cv-significance test of whether the performance of one of these tasks was “cv-significantly” better than that of the other. In particular, task *X* was said to have performance cv-significantly higher than that of task *Y* if and only if $x_i > y_i$ for all ten values of *i*. DeLong divergence $p_{Divg}$ between gene-wide task *G* and subgrouping task *S* was computed by using the predicted mean scores returned by *G* and *S* and the mutation labels of *S* as input for the one-tailed version of DeLong’s test for whether the *S* scores had a significantly better AUC in predicting the labels of *S* than the *G* scores.

### Measuring concordance between subgrouping classifier output and drug response

Summaries of cell line drug response observed within the CCLE cohort as measured by AUC50 were extracted from Table S4B downloaded from the Genomics of Drug Sensitivity in Cancer data portal [98]. Subgrouping classifiers trained on TCGA and METABRIC cohorts were asked to make predictions for the CCLE cohort in the same manner as described for the transfer experiment above. For each combination of drug and task, we thus measured a correlation between subgrouping classifier output and drug response by calculating the Spearman rho between the AUC50 values and the average classifier predictions across the subset of samples for which drug response was available.

## Supplementary information

**Additional file 1: Figure S1.** *Clustering of cohort transcriptomes reveals profiles consistent with molecular subtypes.* We applied unsupervised learning to the expression data used for each cohort considered in this study in order to remove unwanted variation associated with molecular subtypes from our alteration divergence analysis. In conjunction with information on known molecular subtypes present in these cohorts, we identified cases such as METABRIC and TCGA-LGG in which these subtypes clearly overlapped with distinguishable transcriptomic profiles. This contrasted with cohorts such as TCGA-STAD in which subtypes were present but could not be unambiguously linked with unique transcriptomic profiles, and those like TCGA-LUSC in which neither molecular subtypes nor expression clusters were present. The counts of cohort samples with each subtype are listed in the plot legends. In cohorts where molecular subtypes were found to have identifiable transcriptomic profiles we created sub-cohorts that only included samples from a particular subtype or set of subtypes. Unsupervised learning on these sub-cohorts’ transcriptomes revealed that they did not exhibit the large-scale clusters of samples observed in the original cohorts and were thus much more suitable as input for our mutation classification pipelines. We include here these UMAP clusterings for the entire METABRIC cohort and the METABRIC-(LumA) cohort and for the remaining cohorts at our data portal under Figures/S1 - Cohort UMAP Clustering. The names of these figures have the format (expr-source)__(cohort)__UMAP_comps-0_1.svg.

**Additional file 2: Table S1.** *Inventories of subgrouping task information.* For each cohort and sub-cohort in which subgroupings were enumerated, we list here information for the subgroupings of point mutations (Base), the subgroupings constructed by adding CNAs (Copy), as well as the cohort-specific and gene-specific randomly-chosen subgroupings (RandCoh and RandGene). We include here these files for the METABRIC(LumA) cohort: mtype-tbl for an inventory of the subgroupings tested, auc-mat for subgrouping perfomances and Delong divergence relative to the corresponding gene-wide task, coef-means for averaged logistic ridge regression model coefficients, and trnsf-aucs for transfer performances when applied to the remaining cohorts, as well as a README describing the format of each file. These files for the remaining cohorts can be found at our data portal under Datasets/Output Summaries/Ridge.

**Additional file 3: Figures S2.** *Comparing GATA3 subgrouping model coefficients identifies genes implicated in transcriptional divergences.* We compare the model gene coefficients for the gene-wide GATA3 task in METABRIC(LumA) to coefficients for selected GATA3 subgroupings, including the best performing subgrouping as measured by AUC (frameshift_variant-5-6-splice_acceptor_variant–). Genes whose coefficients are in the top ten or bottom according to coefficient value for either model are highlighted in each plot, as are genes in the top twenty-five according to absolute difference between the coefficient values of the two task models. Genes discussed in the text are given bolded labels where applicable. We include here these files for subgroupings of GATA3 cv-significantly better than the GATA3 gene-wide task in METABRIC(LumA) cohort and for similarly significant subgroupings of the remaining genes and cohorts at our data portal under Figures/S2 - Subgrouping Coefficient Comparisons. The names of these figures have the format (expr-source)__(cohort)__(gene)__(subgrouping)__divergence-scatter_Ridge.svg.

**Additional file 4: Figure S3.** *Clustering subgrouping model coefficients reveals structure of mutation heterogeneity.* We applied hierarchical clustering to examine the average regression model gene coefficients across all forty cross-validation folds for each of our subgrouping tasks. Subgroupings with task AUCs of below 0.7 were omitted, as were genes that did not have an absolute model coefficient ranked in the top five for any of the remaining tasks. Distances between subgroupings were computed by taking the inverse of the Spearman correlation across all gene coefficients; these were then used to cluster subgroupings into five groups. To facilitate presentation, here we only show these clusterings for subgroupings which did not have another subgrouping in the same cluster with a higher AUC and a Jaccard index of at least 0.9 with respect to the subgroupings’ mutated samples. The subgroupings with the highest AUC in each cluster are bolded, as is the gene-wide task. An asterisk is placed next to the AUCs of subgroupings with cv-significantly better performance than that of the gene-wide task. We include here these heatmaps for GATA3, TP53, and PIK3CA in METABRIC-(LumA) as well as KRAS in TCGA-LUAD. The corresponding figures for the remaining cases can be found at our data portal under Figures/S3 - Gene Coefficient Heatmaps. The names of these figures have the format (expr-source)__(cohort)__(gene)_auto-heatmap_Ridge.svg.

**Additional file 5: Figure S4.** *Subgrouping prediction tasks outperform cohort-specific random background prediction tasks.* For each of the learning tasks completed in METABRIC(LumA) to predict the presence of an actual mutation or subgrouping of mutations, five additional tasks were performed based on predicting a simulated set of point mutations. These sets were constructed by randomly selecting a group of samples from the entire cohort of the same size as the group of samples affected by the original “real” mutation in the cohort. In addition to these cohort-specific random subgroupings, we also constructed five gene-specific random subgroupings for each task by selecting samples as above using only those samples carrying any point mutation of the gene associated with the task’s subgrouping. (left) We compare the AUCs of cohort-specific random subgroupings to the AUCs of the original subgroupings. (right) We compare the AUC of each original subgrouping to the best AUC across the gene-specific random subgroupings of the same number of “mutated” samples. We include here these figures for the METABRIC-(LumA) and TCGA-BRCA(LumA) cohorts and for the remaining cohorts at our data portal under Figures/S4 - Cohort Null Background AUC Comparisons. The names of these figures have the format (expr-source)__(cohort)__cohort-comparison_Ridge.svg.

**Additional file 6: Figure S5.** *Subgrouping prediction tasks outperform gene-specific random background prediction tasks.* In addition to our cohort-specific null background tasks, we also constructed gene-specific random tasks for each tested subgrouping by randomly selecting five size-matched groups from the set of samples carrying any point mutation of the gene in the cohort. The AUCs of these gene-specific random tasks (grey distributions) tended to be lower than the AUCs of subgroupings (colorful distributions) in genes that had at least one subgrouping task with an AUC of 0.7. Distributions of subgroupings containing at least one subgrouping with performance cv-significantly better than that of the gene-wide task (dashed line) are annotated with an asterisk; genes in which at least one random subgrouping was cv-significantly better than the best original subgrouping are also annotated with an asterisk. We include here these figures for the METABRIC-(LumA) and TCGA-BRCA(LumA) cohorts and for the remaining cohorts at our data portal under Figures/S5 - Gene Null Background AUC Comparisons. The names of these figures have the format (expr-source)__(cohort)__gene-comparisons_Ridge.svg.

**Additional file 7: Figure S6.** *Increasing computational complexity does not change or improve upon classification performance.* We observed similar subgrouping classification performance in METABRIC(LumA) when we repeated our prediction tasks with (**a**) a support vector machine classifier and (**b**) a random forest classifier in place of the logistic ridge regression classifier that was originally used. (**c**) Using these more computationally complex classifiers did not result in improved classification performance across all non-random classification tasks, nor did tuning using a greater number of potential hyper-parameter values.

**Additional file 8: Figure S7.** *Applying an expanded search space to subgrouping enumeration and classification in METABRIC(LumA).* The task enumeration step in METABRIC(LumA) was modified to allow for subgroupings of up to three branches each containing at least five samples for a total of at least twenty samples. This resulted in an expanded search space of 6483 subgroupings. (**a**) The AUCs of the optimal subgroupings found for each gene are shown in the same style as in Fig. 1. (**b**) The AUCs of all subgroupings enumerated using the original search criteria (left) and the additional subgroupings enumerated using the expanded search criteria (right). Distributions of subgroupings containing at least one subgrouping with performance cv-significantly better than that of the gene-wide task are annotated with an asterisk; genes in which at least one subgrouping enumerated using the expanded criteria was cv-significantly better than the best original subgrouping are also annotated with an asterisk.

**Additional file 9: Figure S8.** *Adding copy number alterations to subgrouping classifiers.* We augmented our classification tasks by adding deep amplifications and deep deletions to each subgrouping where there were at least five of one of these two types of mutations present in the corresponding gene within the cohort. Here we compare the classification performance of the best found subgrouping containing CNAs (y-axis) to the gene-wide task (x-axis) for each cancer gene with enumerated subgroupings in each cohort. We include here these figures for the METABRIC-(LumA) and TCGA-BRCA(LumA) cohorts and for the remaining cohorts at our data portal under Figures/S8 - CNA Subgrouping AUC Comparisons. The names of these figures have the format (expr-source)__(cohort)__copy-comparisons_Ridge.svg.

**Additional file 10: Figure S9.** *Subgrouping classification tasks preserve their efficacy when transferred across breast cancer cohorts.* We asked the logistic ridge regression models trained to predict mutation subgroupings in METABRIC(LumA) to make predictions using the TCGA-BRCA(LumA) expression data (top row), and likewise using trained TCGA-BRCA(LumA) models and METABRIC(LumA) expression data (bottom row). The transferred models for the best found subgroupings were successful in recapitulating their original performance relative to the corresponding gene-wide tasks (left column) in the transfer setting (right column).

**Additional file 11: Figure S10.** *Subgrouping behavior replicates across various choices of breast cancer expression datasets.* We observed subgrouping classification performance similar to that in METABRIC(LumA) and TCGA-BRCA(LumA) when we repeated our prediction tasks using (**a**) kallisto TPM expression calls instead of Firehose RSEMs in TCGA-BRCA(LumA), (**b**) both luminal subtypes present in METABRIC, (**c**) all nonbasal subtypes present in METABRIC, (**d**) both luminal subtypes present in TCGA-BRCA (**e**) all nonbasal subtypes present in TCGA-BRCA.

**Additional file 12: Figure S11.** *Divergent subgrouping behavior is present in many cancer cohorts.* We repeated the subgrouping enumeration and classification experiment to characterize alteration divergence across fourteen TCGA cohorts in addition to TCGA-BRCA as well as METABRIC and Beat AML. The AUCs of the best found subgrouping task for each gene are compared here to the corresponding gene-wide task in the same style as Fig. 1. We include here these figures for the TCGA-BLCA and TCGA-HNSC(HPV-) cohorts and for the remaining cohorts at our data portal under Figures/S11 - Subgrouping AUC Comparisons by Cohort. The names of these figures have the format (expr-source)__(cohort)__sub-comparisons_Ridge.svg.

**Additional file 13: Figure S12.** *Comparing cancer gene mutation landscape characteristics across tumor contexts.* The best subgroupings in each cohort for all genes with at least one classification task with an AUC of at least 0.7 in two of the cohorts considered in this study. Each pie chart in a panel represents a cohort in which subgrouping mutation classifiers were trained and tested for the gene in question, with pie charts scaled, sliced, and labelled according to the same schema as in Fig. 1. We include here these figures for TP53, PIK3CA, and NFE2L2 and for the remaining genes at our data portal under Figures/S12 - Subgrouping AUC Comparisons by Gene. The names of these figures have the format (gene)__sub-comparisons_Ridge.svg.

**Additional file 14: Figure S13.** *Transferring mutation signatures across disease contexts.* Models trained to predict the presence of mutations and their subgroupings in each cohort were applied to every other cohort in which the corresponding mutation was also present. (top) For each gene matching the criteria used in Additional file 13: Figures S12, we measured the performance of the gene-wide classifier according to the training cohort (x-axis) and the cohort they were transferred to (y-axis). (bottom) Where applicable, transfer AUC performance of the optimal subgrouping according to most frequent cv-significance relative to the gene-wide task across all cohorts in which subgroupings were enumerated. We include here these figures for TP53, PIK3CA, and NFE2L2 and for the remaining genes at our data portal under Figures/S13 - Gene Transfer AUC Heatmaps. The names of these figures have the format (gene)__transfer-aucs_Ridge.svg.

**Additional file 15: Figure S14.** *Comparing subgrouping divergences across transfer contexts.* Transfer performance of the optimal subgrouping across all cohort pairs for each gene chosen using the same criteria as in Additional file 12: Figures S11. Each pie chart corresponds to an instance of training the gene-wide and best found subgrouping classifiers in one cohort then asking them to make predictions in another cohort. Pie charts are sized according to the proportion of samples carrying any point mutation of the gene in the training cohort, with slices denoting the proportion of mutants belonging to the optimal subgrouping in the training cohort. We include here these figures for PIK3CA, NFE2L2, and RB1 and for the remaining genes at our data portal under Figures/S14 - Gene Transfer AUC Comparisons. The names of these figures have the format (gene)__transfer-comparisons_Ridge.svg.

**Additional file 16: Figure S15.** *Comparing subgroupings against mutation subsets defined by other tools for measuring variant significance.* Classification tasks were created in which the top *n* samples according to the value of various continuous mutation properties were treated as a discrete subgrouping. Using the same training and testing regime as before, we compare the AUCs for these tasks to those for subgrouping tasks created using our original discrete approach. This revealed cases such as EGFR in TCGA-LUAD and NFE2L2 in TCGA-HNSC(HPV-) where using subgroupings was clearly superior to using these metric cutoffs as well as cases such as TP53 and PIK3CA in TCGA-BRCA(LumA) where neither subgroupings nor cutoffs significantly outperform the gene-wide classifier. Legend labels are annotated with an asterisk for classes of subgroupings in which the best subgrouping was cv-significantly better than the original gene-wide task. We include here these figures for the four cases listed above and for the remaining genes and cohorts at our data portal under Figures/S15 - Threshold Subgrouping AUC Comparisons. The names of these figures have the format (cohort)__(gene)__sub-comparison_Ridge.svg.

**Additional file 17: Figure S16.** *Subgrouping classifier scores reveal relationships between subgroupings and other mutations on the same gene.* For genes with at least one subgrouping task with an AUC of at least 0.7 in the cohorts used in this study, we considered the distributions of scores assigned by the best found subgrouping’s classifier to samples with the subgrouping’s mutations (blue violins), samples with point mutations on the same gene but not in the subgrouping (empty grey violins), and samples that are wild-type for point mutations on the gene (filled grey violins). We include here these figures for the METABRIC-(LumA) and TCGA-BRCA(LumA) cohorts and for the remaining cohorts at our data portal under Figures/S16 - Classifier Scores by Mutated Status. The names of these figures have the format (expr-source)__(cohort)__remainder-scores_Ridge.svg.

**Additional file 18: Figure S17.** *Subgrouping classifier scores reveal relationships between mutations within cancer genes.* We dissected the scores returned by our mutation classifiers for mutations within (**a**) NF1 in METABRIC(LumA), (**b**) GATA3 in METABRIC(LumA), and (**c**) GATA3 in TCGA-BRCA(LumA). Within each panel, rows correspond to classification tasks, with the top row showing scores for the gene-wide task and the remaining rows showing the best found subgroupings for the gene in question. Cohort samples are divided across the panel columns according to the type of mutation on the gene they carry, if any. Points and violins with a dark outline denote samples and populations of samples respectively that carried mutations the task had to predict; if a population contained mutated samples that were in the subgrouping as well as samples that were not in it then the samples in the subgrouping are plotted as points within the violin, which contains all samples in the population in every case.

**Additional file 19: Figure S18.** *Subgroupings divergent in performance are often also divergent with respect to association with clinical phenotypes.* Correlations between drug response in cell lines and transferred subgrouping classifier predictions as shown in Fig. 5 were calculated for each subgrouping cv-significantly better than its gene-wide counterpart across all cohorts. We include here these figures for two subgroupings of GATA3 in METABRIC-(LumA) and NFE2L2 TCGA-LUSC respectively not shown in Fig. 5; the figures for the remaining cv-significant subgroupings across all cohorts are at our data portal under Figures/S18 - Drug Response Correlation Comparisons. The names of these figures have the format (expr-source)__(cohort)__(gene)__sub-comparisons__(subgrouping)_Ridge.svg.

## Data Availability

The datasets used and/or analysed during the current study are available at https://osf.io/gr24t/ or from the corresponding author where necessary upon reasonable request. The code used in these analyses is available at https://github.com/ohsu-comp-bio/dryads-research as described in the text.

## Abbreviations

AUC: Area under the receiver operating characteristic curve
CNA: Copy number alterations
cv: Cross-validation
cvSig: Cross-validated task significance
pDivg: Delong’s test *p* value for task divergence

## Data and code availability

All of the code used for data preparation, machine learning analysis, and plot creation was written in Python 3.6 and can be found at https://github.com/ohsu-comp-bio/dryads-research (release: precepts-1a_v2.0.1, DOI: 10.5281/zenodo.4705943) under the dryads-research/experiments/subgrouping_test subdirectory. Task AUCs, mean coefficient values, and transfer task AUCs for classification tasks can be found at our “dryads-research” Open Science Framework data portal (DOI: 10.17605/OSF.IO/GR24T) at https://osf.io/2m47r/. Likewise, supplementary figures not included in the Additional Files can be found at https://osf.io/28vxb/. Assistance with the reproduction of the results presented herein will be provided upon request.
