# Supplementary figures and images for: Systematic interrogation of mutation groupings reveals divergent downstream expression programs within key cancer genes
Source: BMC Bioinformatics. 2021 May 6;22:233. doi: 10.1186/s12859-021-04147-y (PMC8101181; doi:10.1186/s12859-021-04147-y)

training cohort:  
METABRIC(LumA)

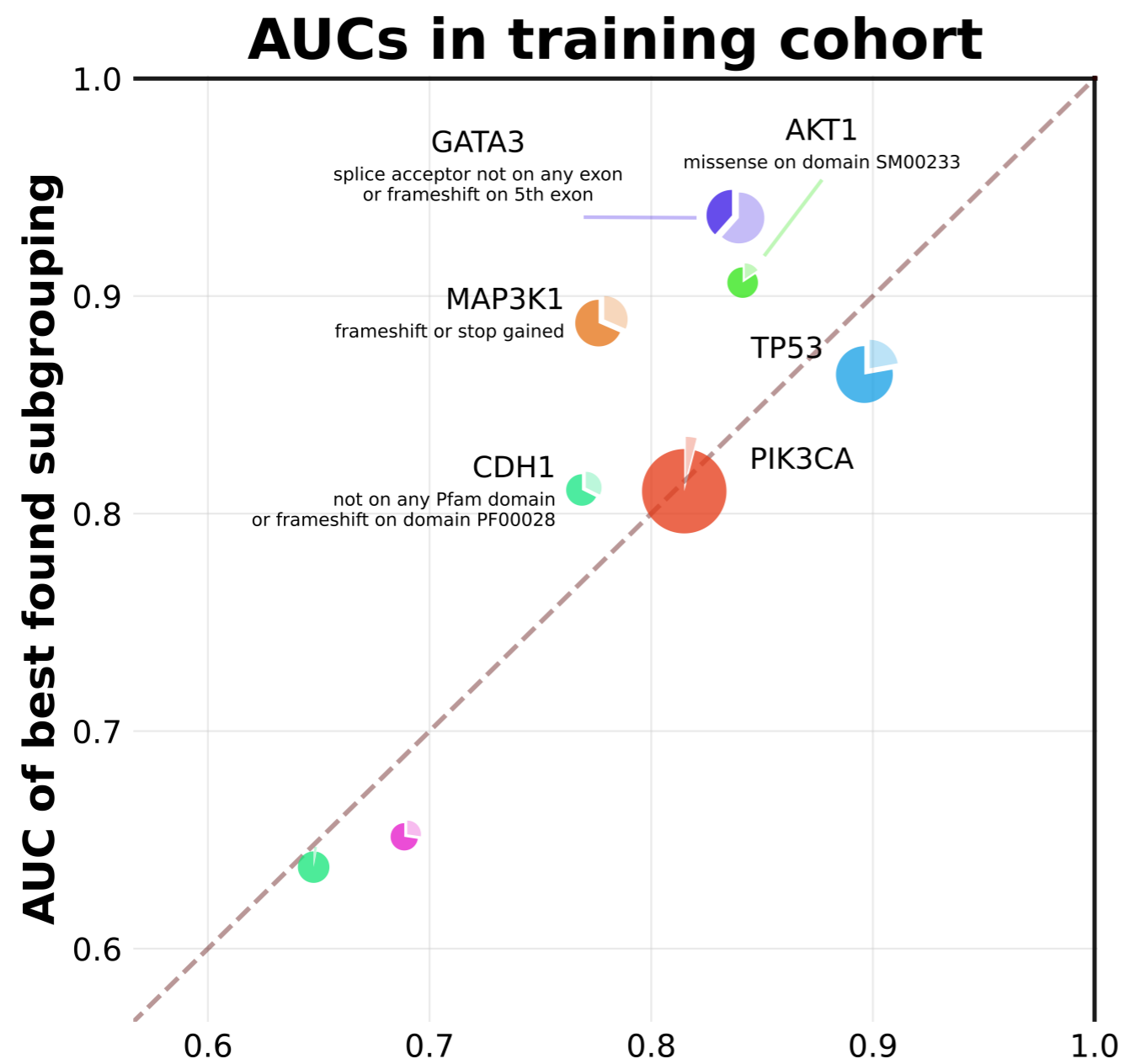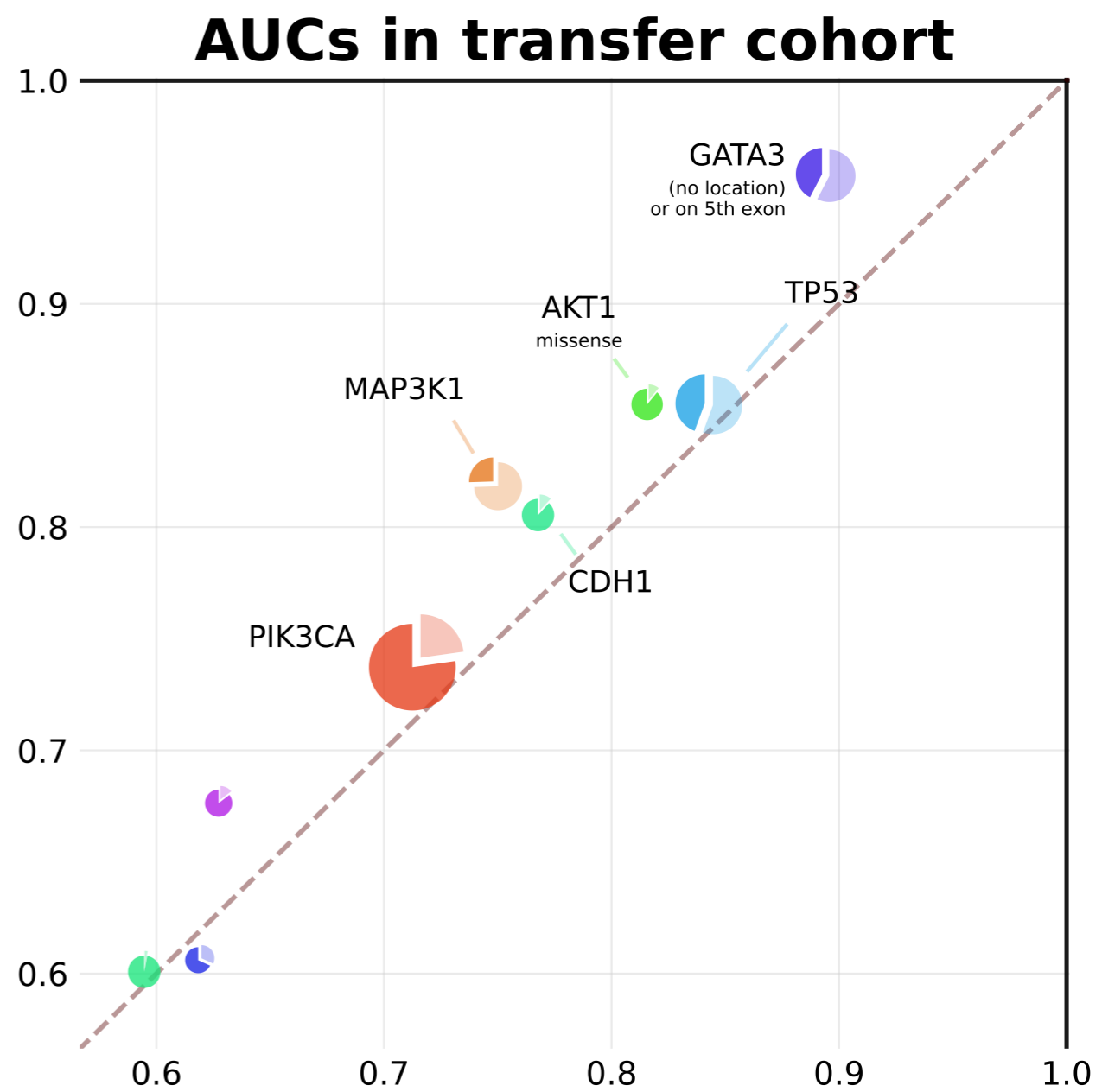

training cohort:  
TCGA-BRCA(LumA)

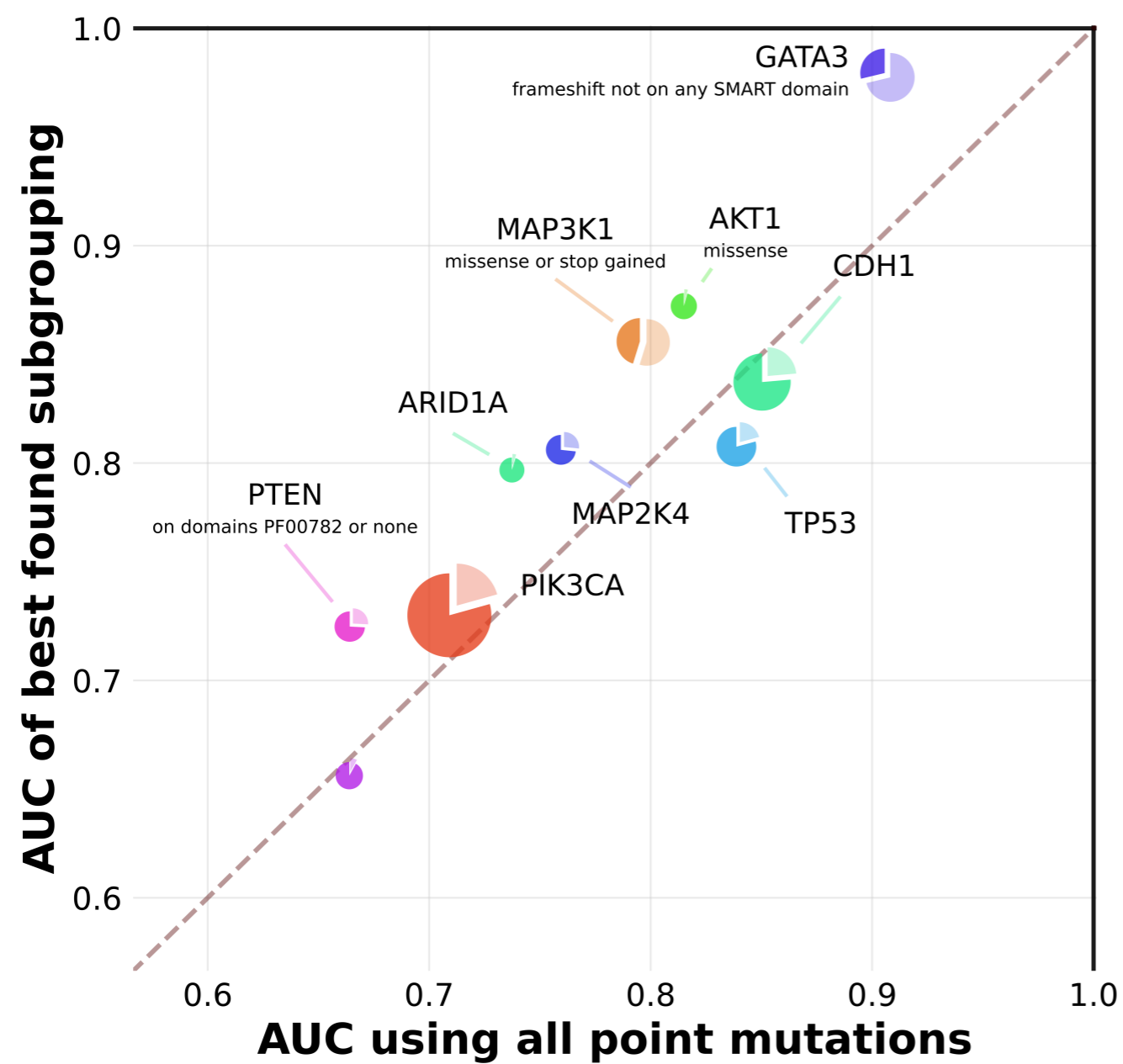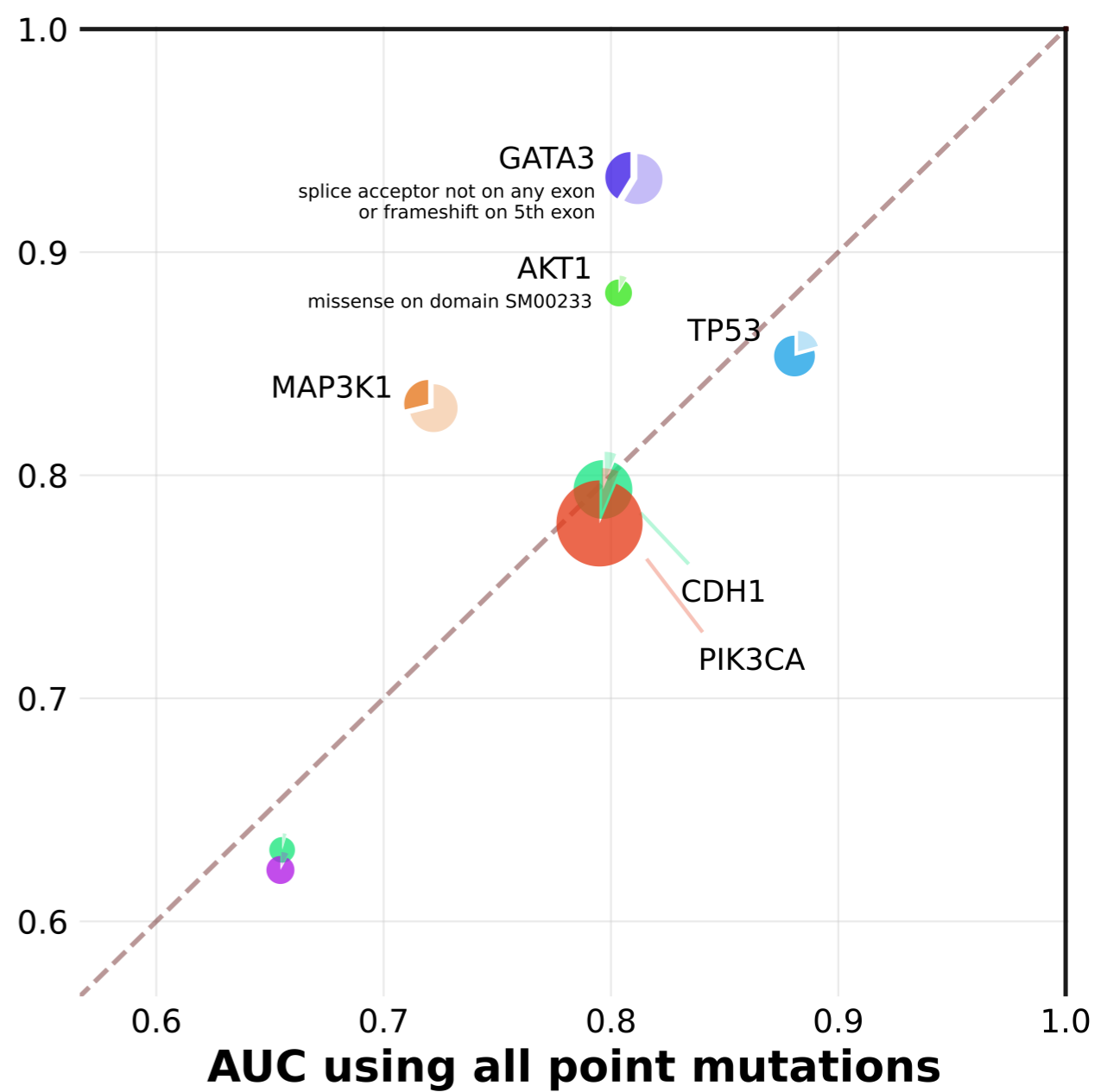

Supplement: Supplementary file 10 — Additional file 10: Figure S9. Subgrouping classification tasks preserve their efficacy when transferred across breast cancer cohorts. We asked the logistic ridge regression models trained to predict mutation subgroupings in METABRIC(LumA) to make predictions using the TCGA-BRCA(LumA) expression data (top row), and likewise using trained TCGA-BRCA(LumA) models and METABRIC(LumA) expression data (bottom row). The transferred models for the best found subgroupings were successful in recapitulating their original performance relative to the corresponding gene-wide tasks (left column) in the transfer setting (right column). [file 12859_2021_4147_MOESM10_ESM.pdf]
